# Supplementary material for: MucD regulates alginate biosynthesis through the proteolytic control of AlgX and AlgK in Pseudomonas aeruginosa
Source: PLoS Biol. 2026 Sep 21;24(9):e3003985. doi: 10.1371/journal.pbio.3003985 (PMC13608001; doi:10.1371/journal.pbio.3003985)
Supplement: Supplementary file 1 [file pbio.3003985.s020.pdf]

Fig. 1A

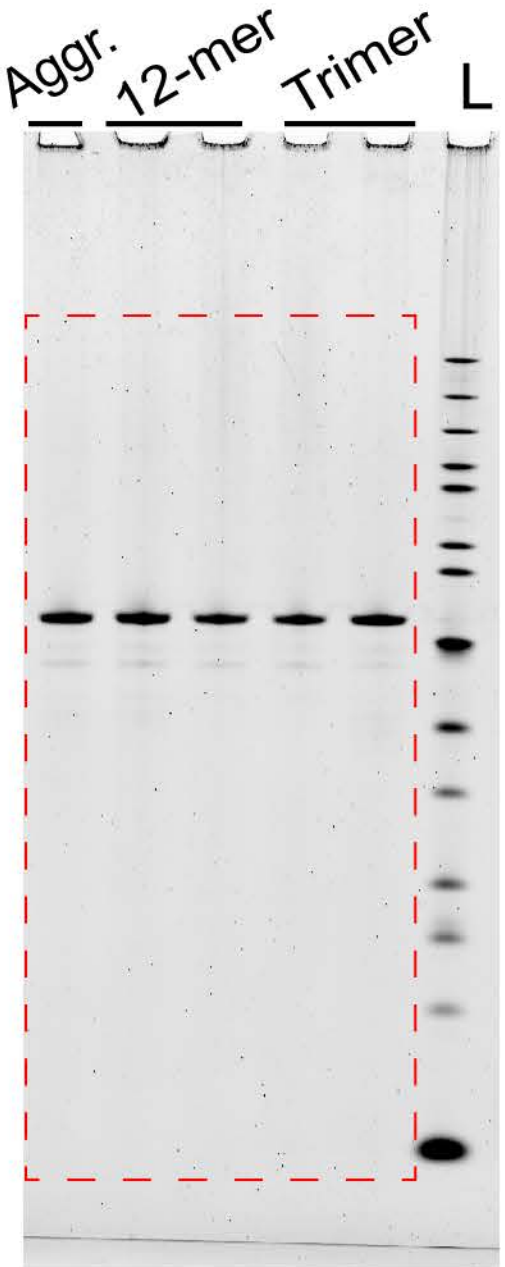

SDS-PAGE
SYPRO Ruby

Fig. 1B

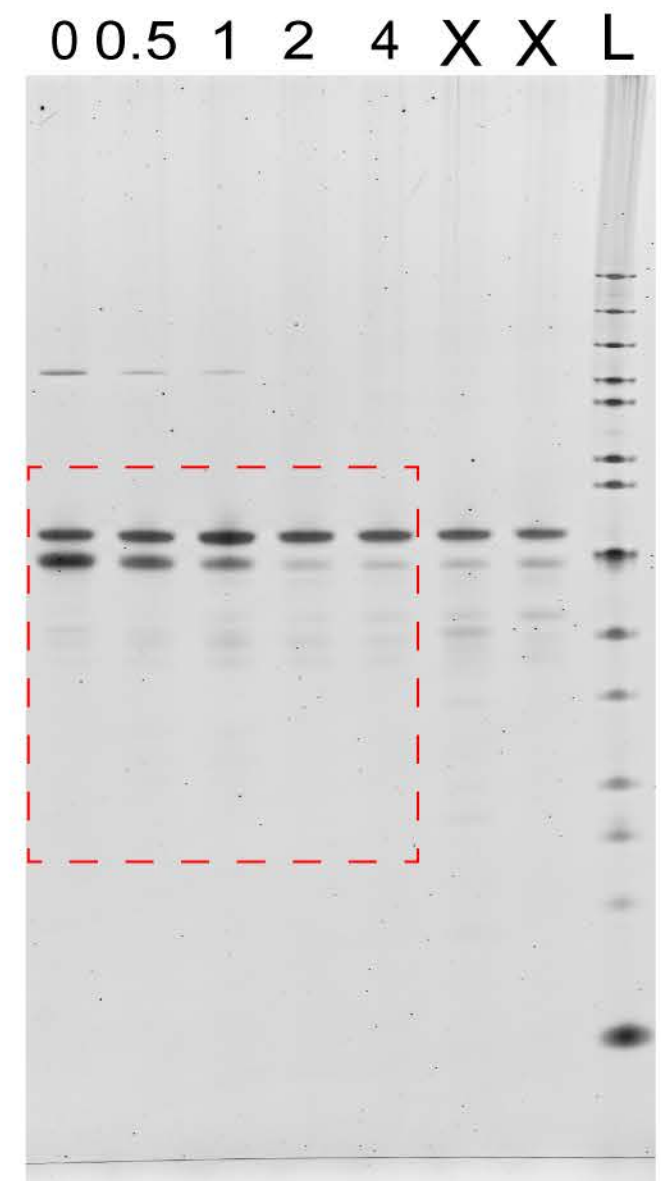

SDS-PAGE
SYPRO Ruby

Fig. 4D

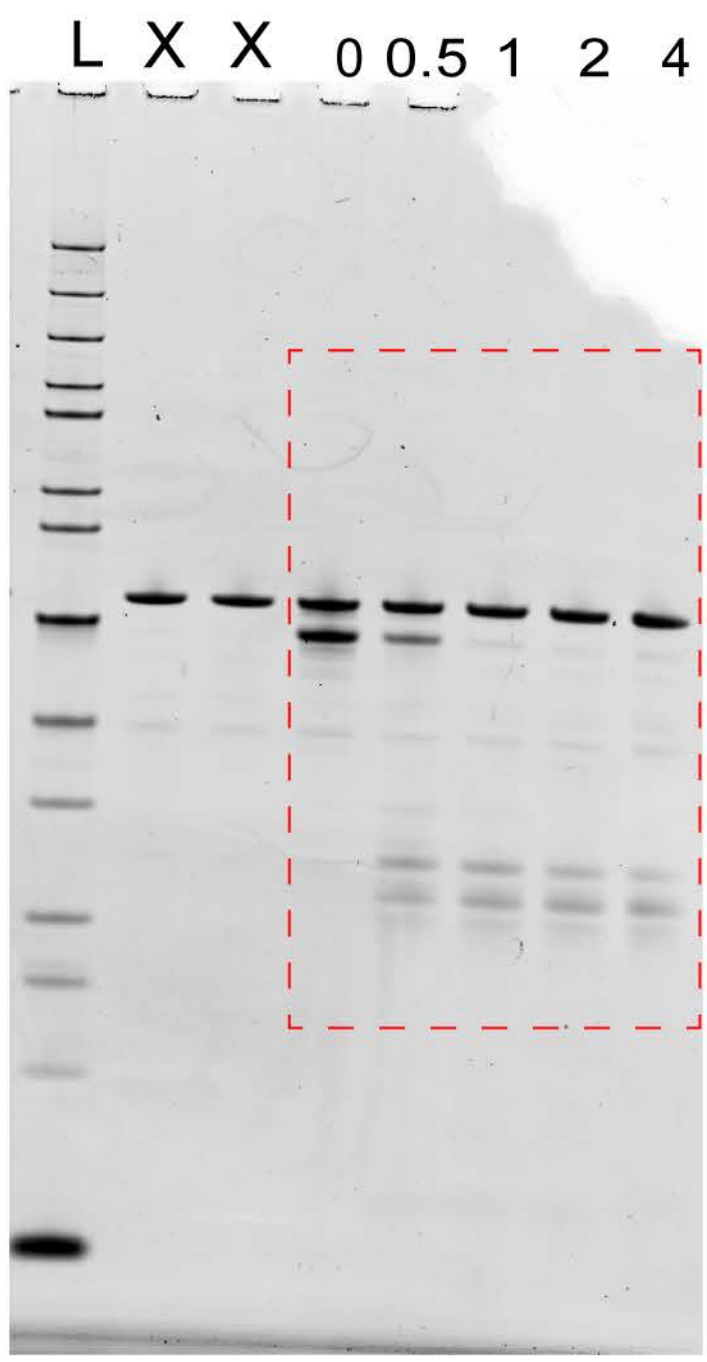

SDS-PAGE
SYPRO Ruby

Fig. 4E

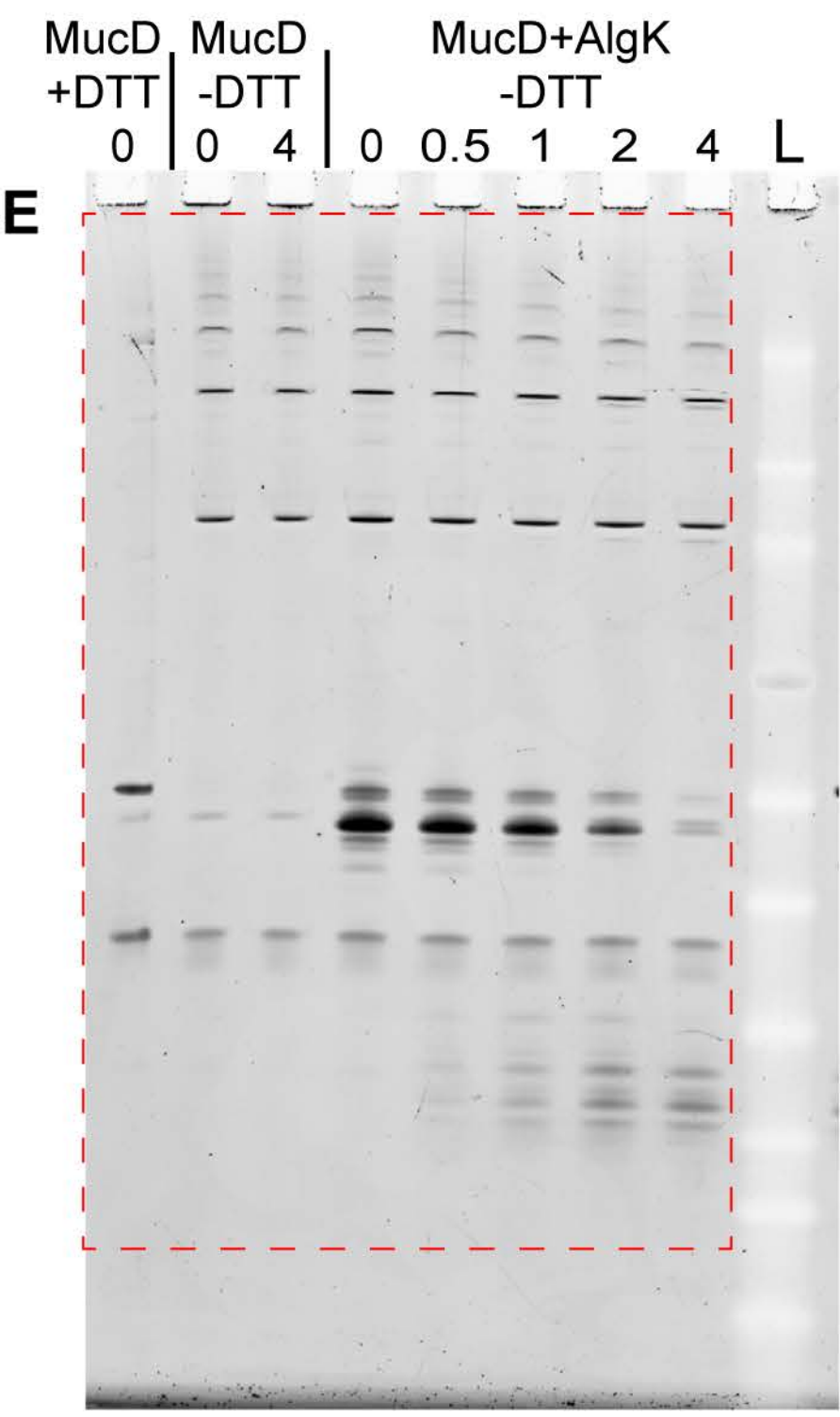

SDS-PAGE
SYPRO Ruby

Fig. S1C

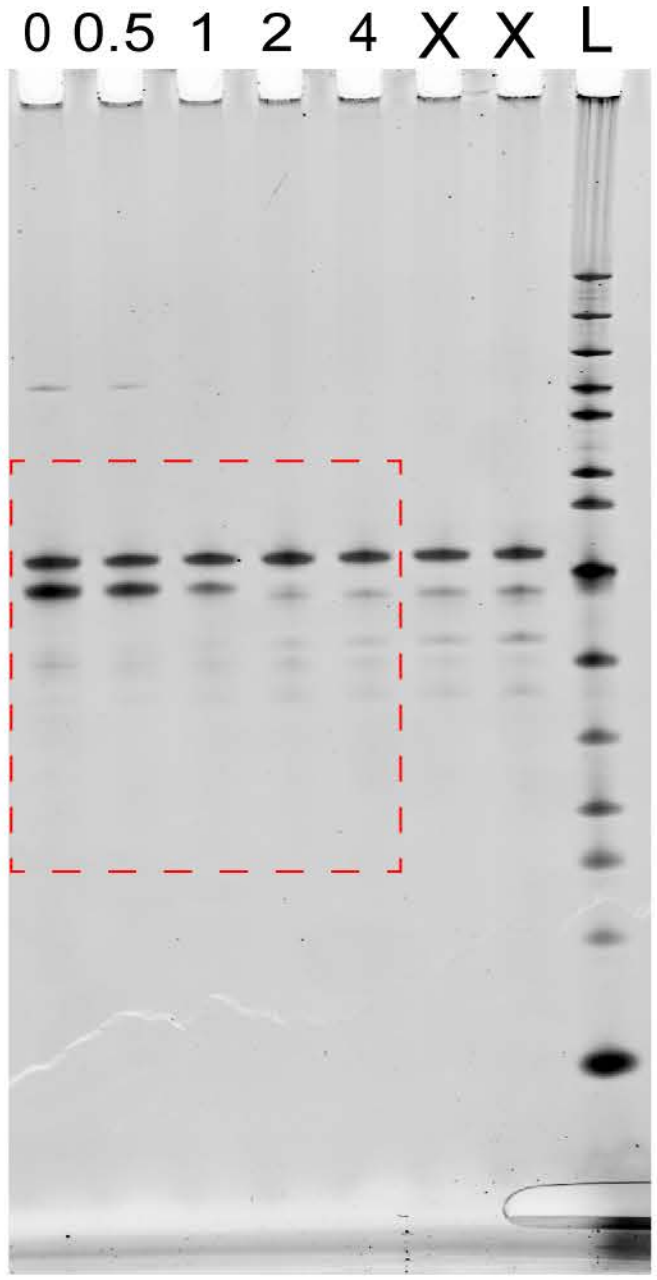

SDS-PAGE
SYPRO Ruby

Fig. S1D

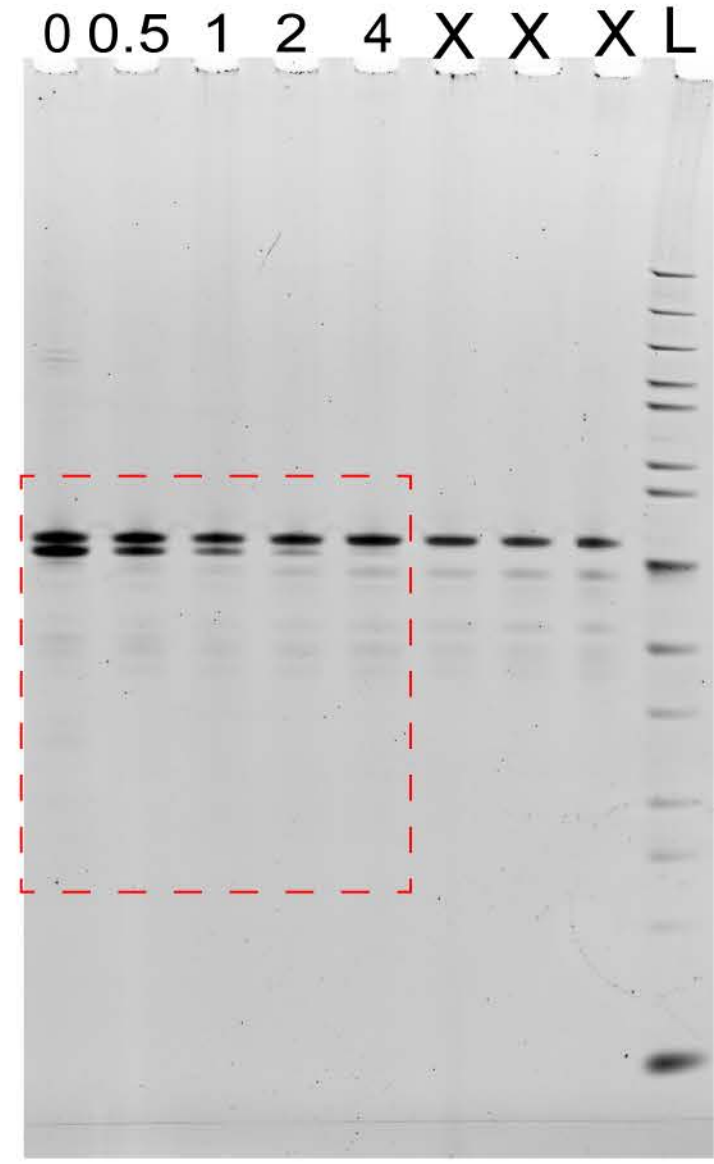

SDS-PAGE
SYPRO Ruby

Fig. S1E

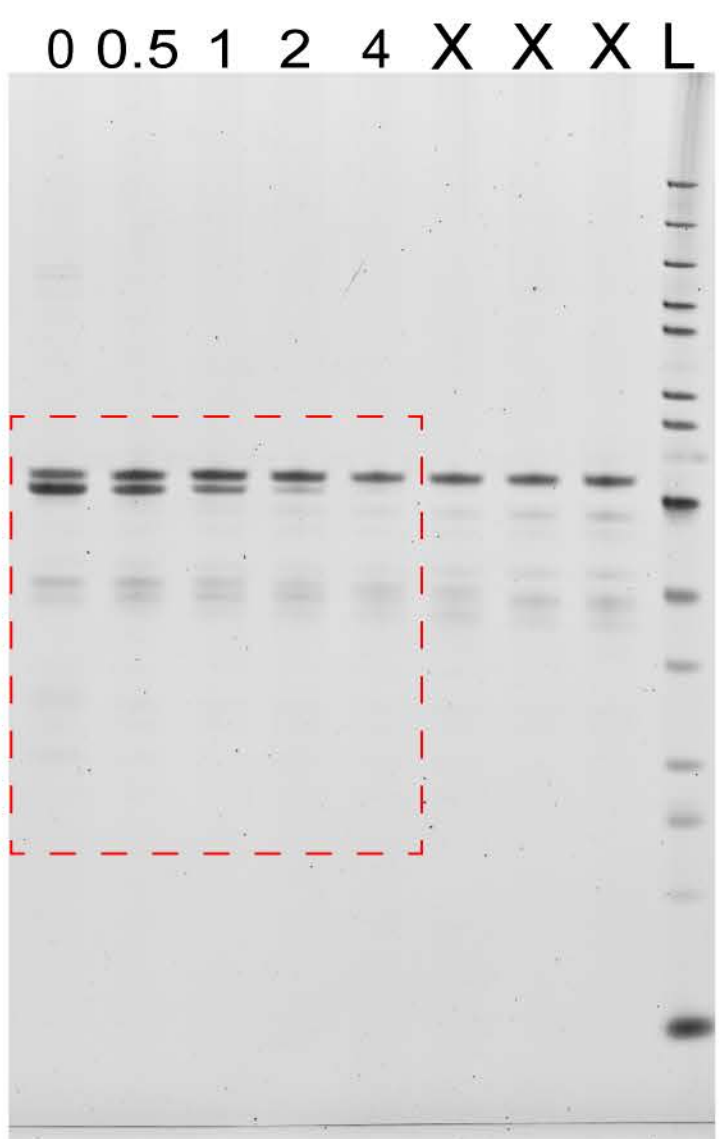

SDS-PAGE
SYPRO Ruby

Fig. S2A

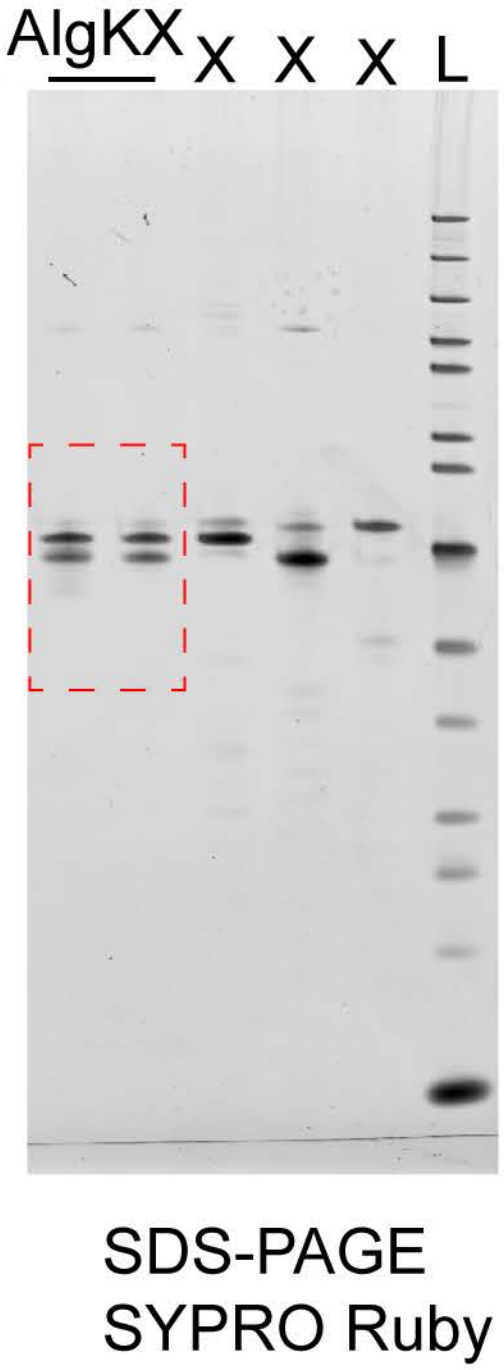

Fig. S2B

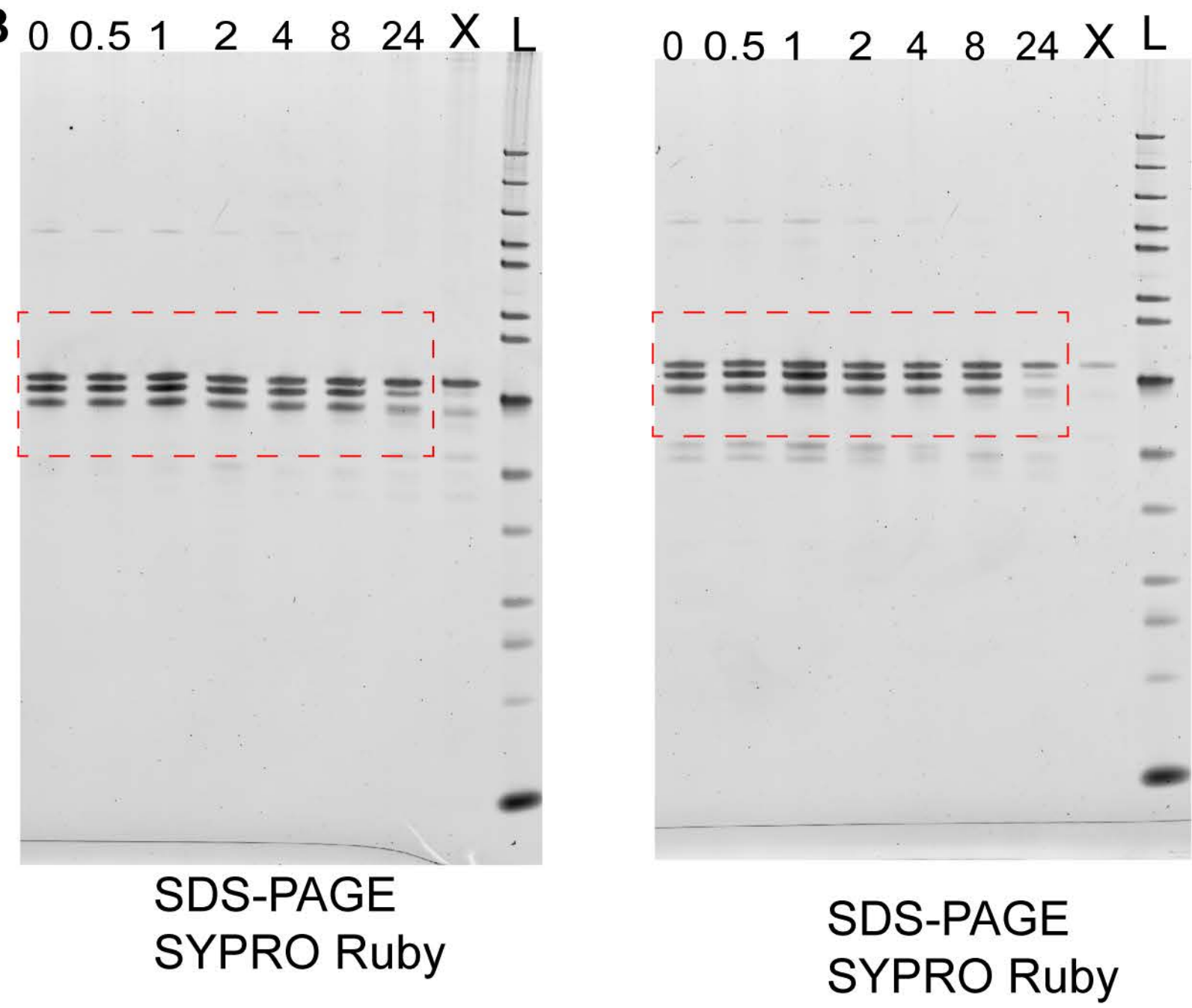

Fig. S5A

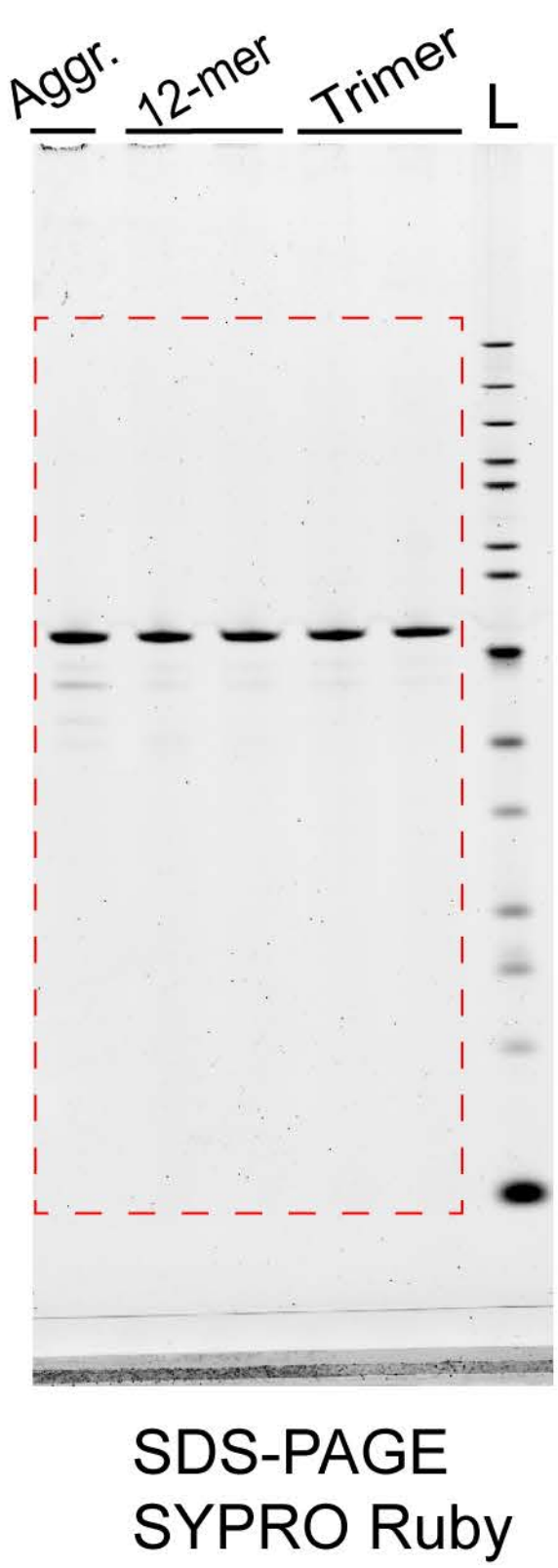

Fig. S5B

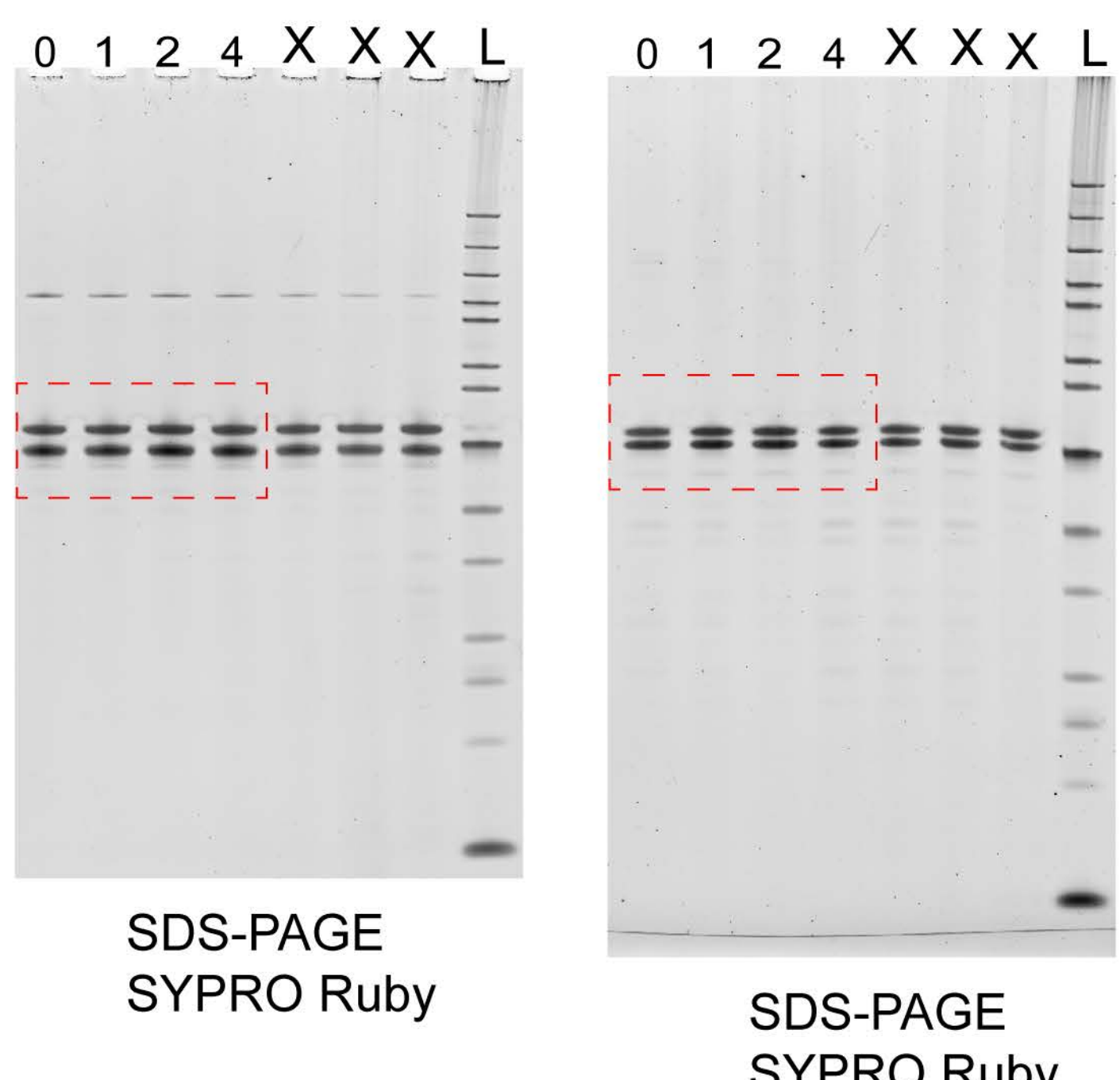

Fig. S5E

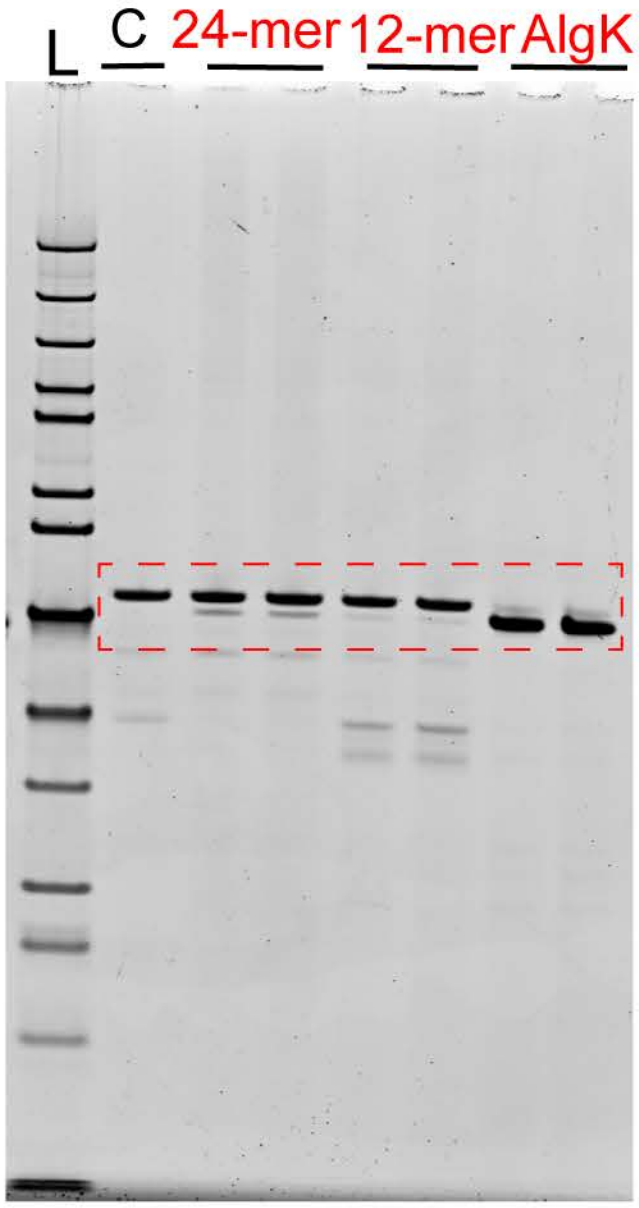

SDS-PAGE
SYPRO Ruby

Fig. S9F

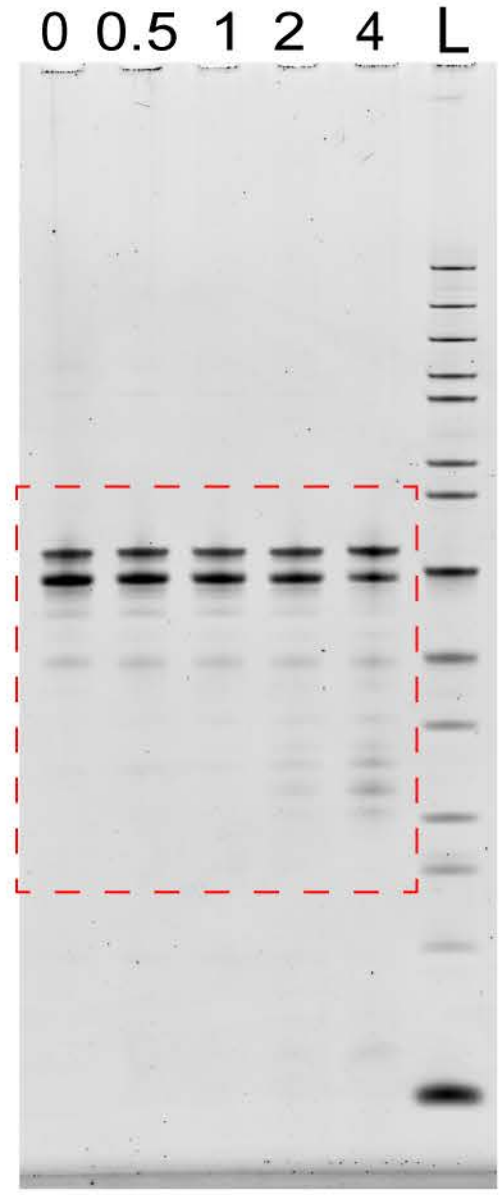

SDS-PAGE
SYPRO Ruby

Fig. S11E

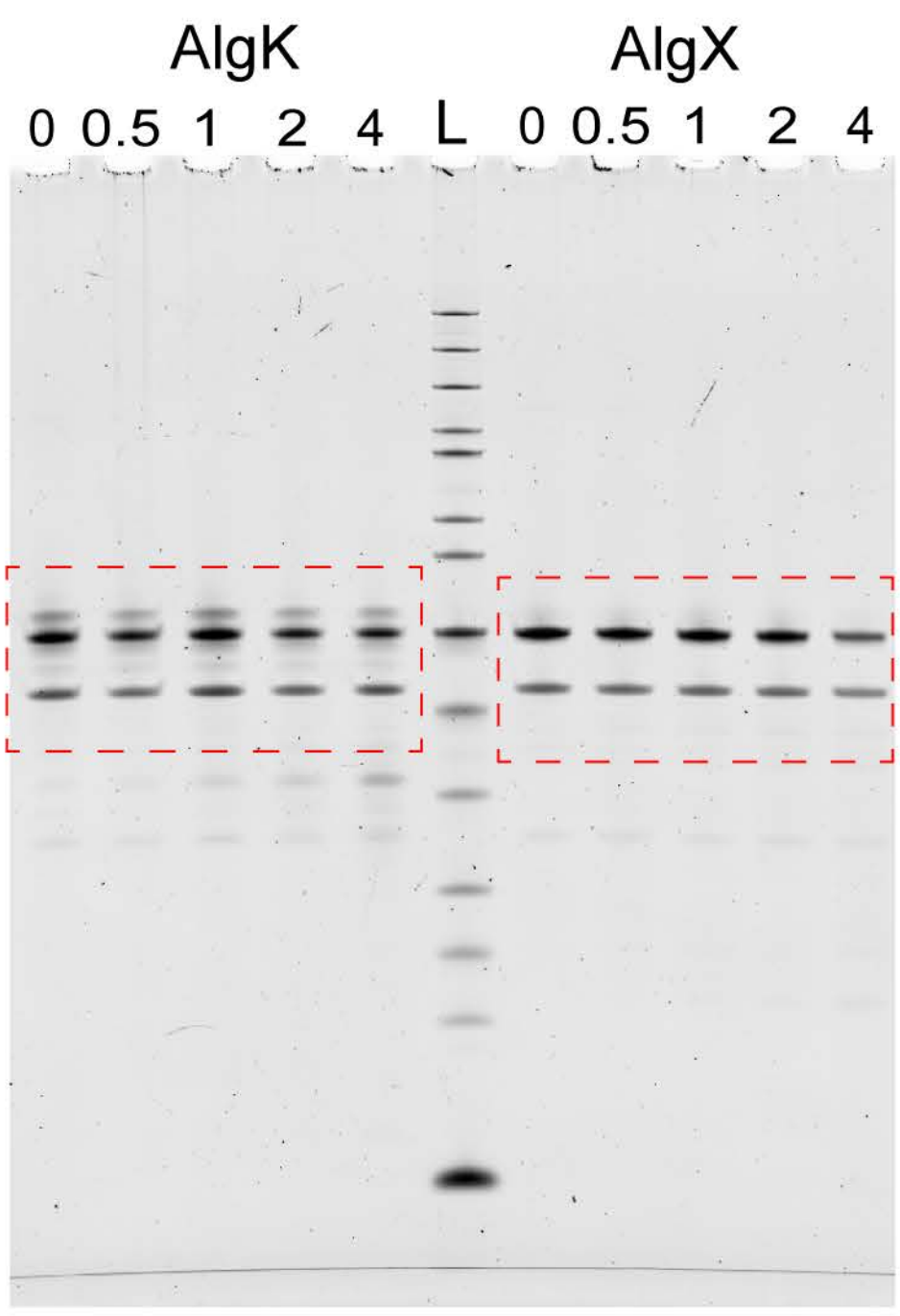

SDS-PAGE
SYPRO Ruby

Fig. S12D

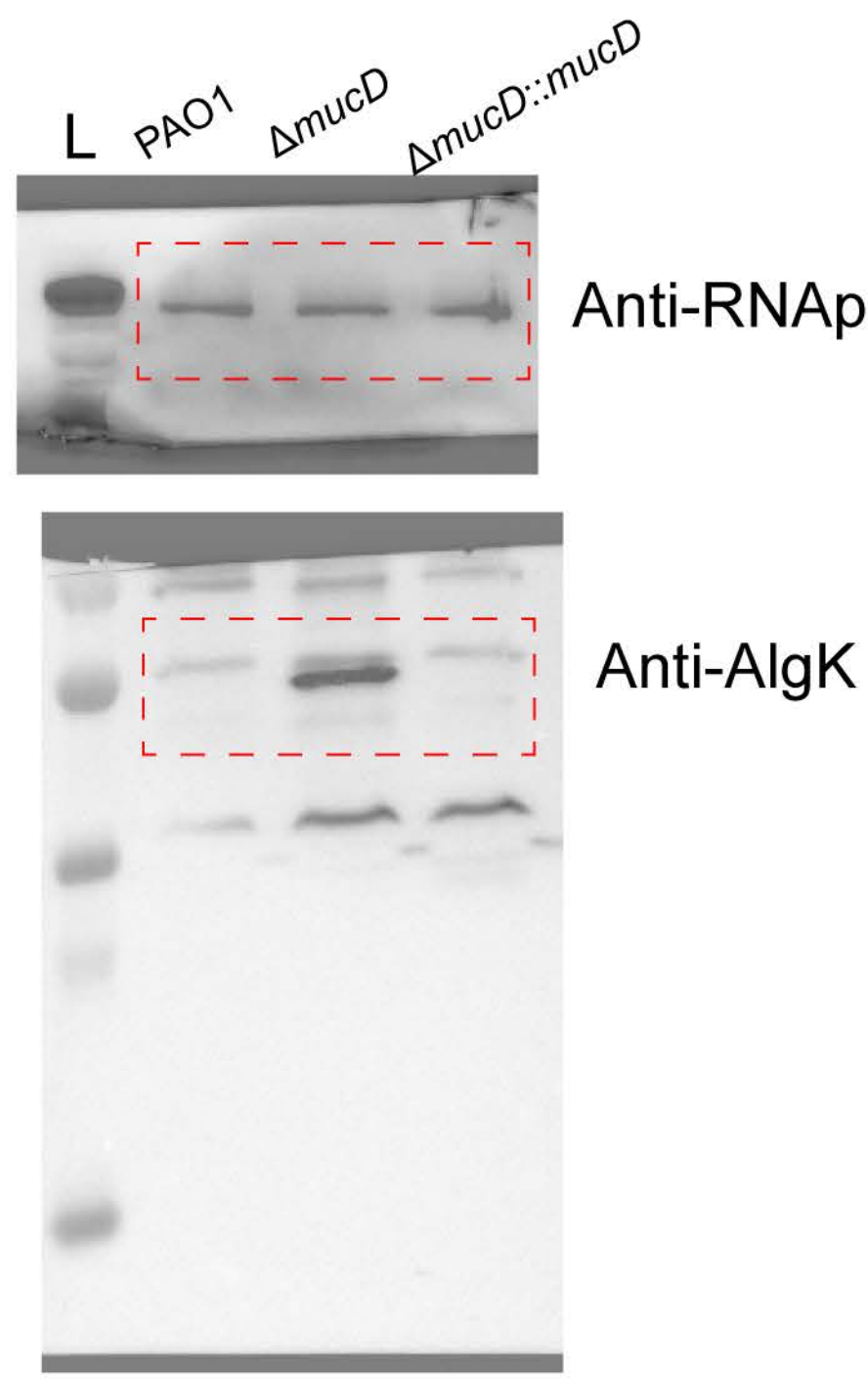

Western Blot

Fig. S13A

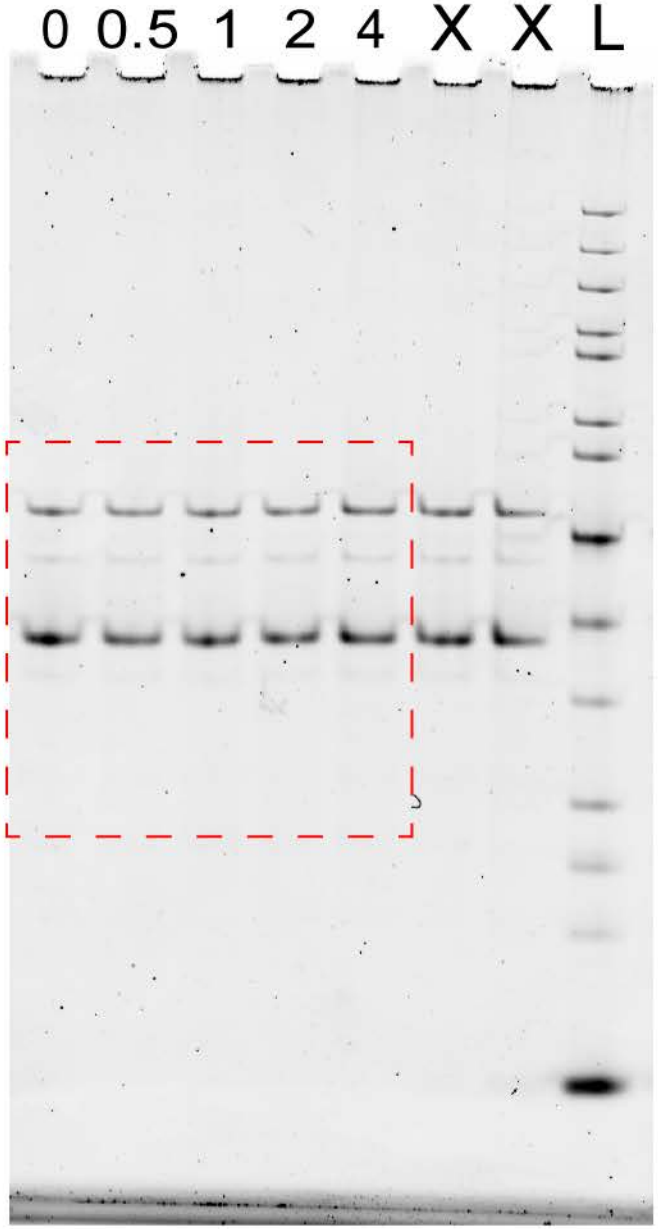

SDS-PAGE
SYPRO Ruby

Fig. S13B

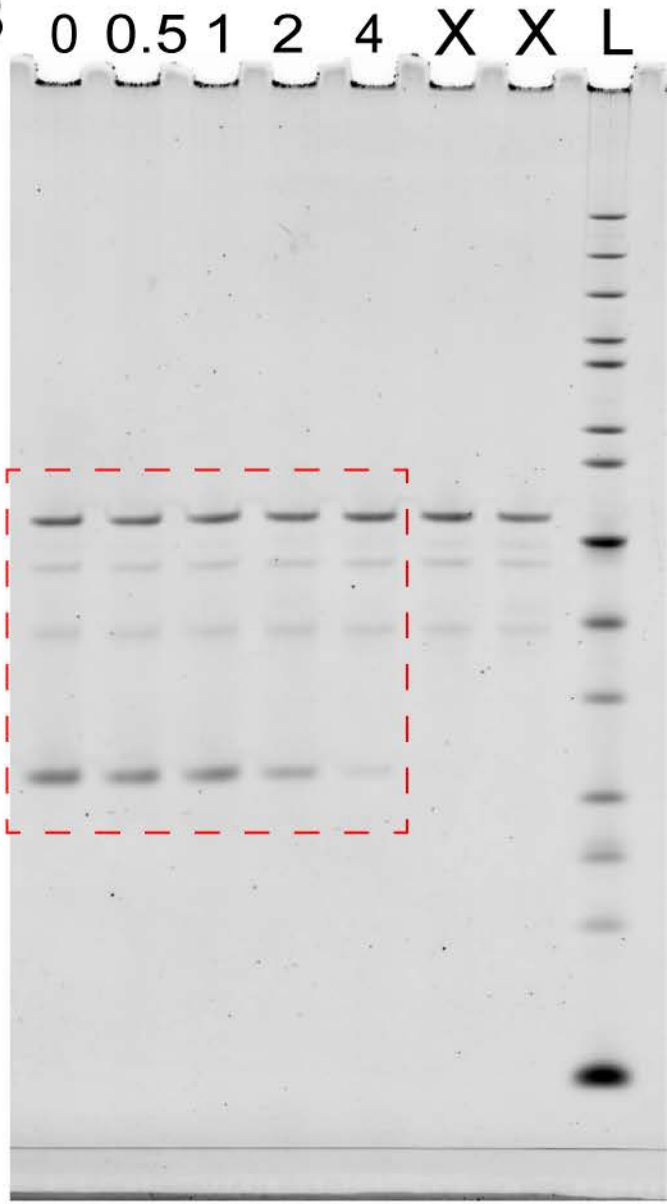

SDS-PAGE
SYPRO Ruby
