# Supplementary material for: MucD regulates alginate biosynthesis through the proteolytic control of AlgX and AlgK in Pseudomonas aeruginosa
Source: PLoS Biol. 2026 Sep 21;24(9):e3003985. doi: 10.1371/journal.pbio.3003985 (PMC13608001; doi:10.1371/journal.pbio.3003985)
Supplement: Supplementary file 3 [file pbio.3003985.s024.docx]

**Point-by-Point Response**

(Our response colored blue)

Reviewer #1: This manuscript presents a structural and biochemical investigation of the HtrA-like protease MucD in Pseudomonas aeruginosa. The authors identified AlgK and AlgX as direct substrates, determined cryo-EM structures of MucD assemblies, and proposed a novel post-translational regulatory mechanism for alginate biosynthesis governed by a trimer-to-12-mer dynamic equilibrium. While the structural biology work is substantial and uncovers an intriguing mechanism distinct from its homolog DegP , the manuscript currently lacks the robust in vivo physiological validation. Specifically, the physiological relevance of the proposed structural models and the in vivo substrate verification require further experimental support. I recommend a Major Revision pending the inclusion of critical supplemental experiments.
>> Thank you for the positive comments, we have addressed the concerns as outlined below.

Major Concerns
1. The claim that AlgK and AlgX are physiological substrates relies heavily on in vitro assays. In vivo evidence is currently limited to an increase in AlgK abundance in the ΔmucD mutant. The authors must provide direct in vivo interaction evidence (e.g., co-IP, in-cell crosslinking, or substrate trapping with the S217A mutant) and examine AlgX protein levels in vivo.

>>We thank the reviewer for raising this important point. To examine AlgX abundance *in vivo* as suggested, we generated a polyclonal anti-AlgX antibody and analysed PAO1 and Δ*mucD* cell lysates. While purified AlgX was readily detected as a positive control, endogenous lysates produced high background and multiple nonspecific bands despite repeated optimization, preventing reliable identification or quantification of endogenous AlgX (Fig. R1). For this reason, we include the result here for the reviewer’s reference but have not incorporated it into the revised manuscript.

In addition, previously published affinity-purification experiments have shown that tagged AlgX co-purified with AlgK and MucD from the periplasmic fraction and all three proteins have been detected by MALDI-TOF/MS (Iain David Hay et al., *Applied Microbiology and Biotechnology*, 2012). We made the following changes in the revised manuscript to soften our original statements:

1. In the “Introduction” (Lines 64–66), we now state that affinity-purification studies as:

“…Affinity pull-down studies further suggested that MucD could form a periplasmic complex with the terminal acetyltransferase AlgX and tetratricopeptide repeat (TPR)-rich scaffold protein AlgK [14]…”

2. In the “Results” (Lines 452-458), we revised the conclusion to distinguish the level of support for the two proteins:

“…Our results revealed that AlgK protein level was markedly increased in the Δ*mucD* mutant and restored by *mucD* complementation (Fig. S12D). Together with its degradation by MucD *in vitro* (Fig. 1B, Fig. S1C) and its reported association with MucD *in vivo* [14], these findings support AlgK as a physiological substrate of MucD. AlgX can also be degraded by MucD *in vitro* (Fig. S1 D and E) and was previously identified in the same periplasmic complex, however, its substrate status *in vivo* requires more experiments with complementary approaches.”

**Fig. R1 Detection of AlgX protein expression levels in PAO1 and *ΔmucD* strains.**

Whole cell lysis was used for the detection of the AlgX protein by Western blot with an anti-AlgX polyclonal antibody. PC (Positive Control): the purified AlgX protein. RNA polymerase β-subunit (RNAp) served as a loading control. Detection of AlgX and RNAp was performed on the same blot. A representative result is shown.

2. The authors engineered a disulfide-locked 12-mer mutant, MucD(D282C-T469C), demonstrating its reduced in vitro activity. To form a complete biological loop, this specific mutant must be tested in the in vivo alginate complementation assay. If the structural model holds true, this locked resting-state mutant should fail to suppress alginate overproduction.

>>We appreciate the reviewer’s constructive suggestion. Following this suggestion, we examined the alginate production levels of the Δ*mucD* strain complemented with the MucD(D282C-T469C) mutant. The locked 12-mer mutant failed to suppress alginate overproduction, yielding alginate levels similar to the Δ*mucD* strain. We have integrated the data into Fig. S12A and updated the “Result” section (Lines 433-435) accordingly:

“…In contrast, complementation with either the proteolytically inactive MucD(S217A) variant or the disulfide-locked 12-mer MucD(D282C-T469C) variant did not significantly reduce alginate production (Fig. S12A) …”
3. A significant discrepancy exists regarding oligomerization: full-length substrates induce the active trimer , whereas the synthetic short peptide (AlgK369-388) induces a 24-mer, which was subsequently used for cryo-EM. The authors must clarify and discuss whether this peptide-bound 24-mer represents an authentic physiological recognition event or a post-cleavage intermediate/artifact.

>> We thank the reviewer for this perceptive question regarding the physiological relevance of the higher-order 24-mer assembly. To clarify our observations: for wild-type MucD, incubation with full-length substrate AlgK shifted the oligomeric equilibrium toward the trimer; for the inactive MucD(S217A) mutant, incubation with either the full-length substrate AlgK or the synthetic short peptide AlgK_369-388_ triggered the disappearance of the trimer, and reassembly into the highly ordered 12-mer and 24-mer states, enabling structural determination at atomic resolution by cryo-EM. Notably, both 12-mer and 24-mer structures share the similar substrate-binding pattern and building blocks of trimer.

We have not captured the 24-mer assembly of wild-type MucD *in vitro* or *in vivo*. However, we cannot rule out the possibility that the 24-mer exists *in vivo* under certain conditions, for example representing a post-cleavage intermediate as suggested by the reviewer. We have rephrased the “Discussion” section in the revised manuscript (Lines 511-518) to cover this possibility:

“Interestingly, substrate addition to the proteolytically inactive MucD(S217A) mutant shifted the equilibrium toward 12-mer and 24-mer assemblies, a behavior distinct from that of wild-type MucD (Fig. 1, Fig. S5 E and F). This finding raises the possibility that inhibitory peptides or small proteins, analogous to naturally produced serine protease inhibitors such as serpins [39], could regulate MucD by stabilizing the higher-order oligomeric states as post-cleavage intermediates. Ecotin, a broad-spectrum periplasmic serine protease inhibitor found in many bacteria, including *E. coli* and *P. aeruginosa*, provides one example of this type of post-translational regulation [40].”

We have also discussed the possibility that the 24-mer assembly represented a highly organized chaperone cage in lines 518-526:

“…Although we did not observe 24-mer formation for wild-type MucD, the 12-mer and 24-mer cage assemblies may also relate to a potential chaperone-like function. Under stress conditions, membrane or periplasmic proteins with critical function may be recognized by MucD and trigger the cage formation. Since the higher oligomers of MucD adopt a more closed formation (Fig. S6A), they may shield and stabilize captured substrates from the disordered periplasm environment. However, we did not detect proteins co-purifying with MucD, and no putative chaperone partners have yet been reported. Given that the chaperone activity is highly condition-dependent, whether MucD has chaperone activity remains an open question.”
4. The Congo red assay used to measure EPS is non-specific and can bind to other extracellular polysaccharides (Pel, Psl) and matrix components. To robustly support the central physiological conclusion, the authors must utilize an orthogonal, alginate-specific assay.

>> We appreciate this valuable suggestion from the reviewer to improve the specificity and rigor of our phenotypic data (please also see our response for reviewer 2, Comment 1).

Following the reviewer's advice, we tested the alginate production using the highly established carbazole assay for alginate quantification (Andreea A. Gheorghita et al., *Nature Communications*, 2022). The quantitative results from the carbazole assay are consistent with our Congo red assays, corroborating the same trends in alginate production among the tested strains.

We have updated Fig. 5 (now Fig. S12 due to number limits of main figures for a “Short Reports” type manuscript) and revised the corresponding “Results” section (Lines 425-427):

“…To determine whether this regulation depends on MucD protease activity, we measured alginate-producing levels in Δ*mucD* mutants in the PAO1 background using the carbazole assay, a quantitative method for detecting alginate in *P. aeruginosa* [19,32]…”

We also revised the related “Methods” section to describe the carbazole assay protocol accordingly.

5. The conclusion that MucD represses alginate production primarily via post-translational degradation, independent of transcription, is overstated. Unchanged algK/algX mRNA levels do not preclude transcriptional regulation of other operon genes (such as the rate-limiting enzyme algD) or broader AlgU-dependent effects. The authors should moderate these claims and comprehensively discuss potential crosstalk between these pathways.

>> We agree with the reviewer on the complexity of the *alg* operon's regulation. To address this, we examined the mRNA levels of *algK*, *algX* and *algD* by RT-qPCR in *mucD* related variants, which is also suggested by the third reviewer. While *algK* and *algX* mRNA levels remained unchanged, the transcription of *algD*—the first gene encoding a rate-limiting enzyme in the operon—was significantly increased in the Δ*mucD* strain (Fig. S12C). We have revised the “Result” section accordingly to address these findings and provide possible explanations (Lines 438-448):

“A previous study reported that deletion of *mucD* in PAO1 increases promoter activity of the *alg* operon [14]. To assess whether the elevated alginate production observed here was accompanied by transcriptional changes in *algK and algX*, quantitative reverse transcription PCR (RT-qPCR) for the wild-type, Δ*mucD,* and Δ*mucD*::*mucD* strains was carried out. The results showed that the mRNA levels of *algK* and *algX* did not differ significantly among the strains. By contrast, *algD*, the proximal gene in the operon encoding the rate-limiting enzyme of the pathway, increased ~2.8-fold in the Δ*mucD* strain and restored to wild-type levels upon *mucD* complementation (Fig. S12C). The different behavior of the proximal *algD* transcript and the distal *algK* and *algX* transcripts likely reflects natural transcriptional attenuation or differential mRNA stability across this large 12-gene biosynthetic cluster, consistent with previous evidence for increased promoter activity in Δ*mucD* cells [14,35,36]…”

We have also revised the “Discussion” section (Lines 551-556) to highlight this dual-layer regulation:

“…In large bacterial operons such as the *alg* operon, polycistronic transcripts can undergo progressive downstream attenuation, internal termination, or segmented mRNA decay, uncoupling steady-state transcript levels of distal structural genes (*algK* and *algX*) from primary promoter activity [34]. Collectively, these findings indicate that MucD regulates alginate biosynthesis through both negative transcriptional control of the *alg* operon and post-translational quality control of key assembly components, including AlgK and AlgX…”

6. The proposed substrate-induced 12-mer-to-trimer conversion relies primarily on SEC analysis, which reflects equilibrium states rather than dynamic transitions. The authors are encouraged to provide quantitative enzymatic analyses to strengthen the rigor of this conclusion.

>> We thank the reviewer for the comment and agree that using the term "conversion" implies a dynamic process that our SEC data cannot definitively prove, while performing these quantitative enzymatic analyses is beyond our current technical setup.

To ensure scientific rigor, we have revised our phrasing from "substrate-induced 12-mer-to-trimer conversion" to "…substrate binding shifts the MucD oligomeric equilibrium toward the trimer…" (Lines 110-112).

Minor Concerns

1. The assertion that MucD lacks chaperone activity based solely on the absence of co-purified proteins is unconvincing, as such activity is highly condition-dependent. This statement should be softened or revised.
 >>We have rephrased the relevant section (Lines 94-95) in the revised manuscript to avoid overstating:

“…Furthermore, no convincing additional protein co-eluting with MucD was detected based on SDS-PAGE (Fig. 1A) or MALDI-TOF/MS (Table S1)…”.

2. Figure 5C lacks densitometric quantification. Furthermore, utilizing a cytoplasmic protein as a loading control for periplasmic proteins may not account for extraction efficiency differences or periplasmic-specific leakage. Please justify this choice or use a periplasmic control.

>> We have quantified the AlgK protein bands as the reviewer suggested and modified Figure 5C (now Fig. S12D) accordingly. A *mucD* complementary strain was also included as a control (please also see our response for reviewer 3, “Major Concerns” 11).

While we acknowledge that a periplasmic marker would more precisely address compartment-specific variations, we do not have a reliable, validated antibody for a periplasmic protein as the loading control. Similarly, RNA polymerase β-subunit has been used as the loading control for the detection of periplasmic alginate synthesis proteins (such as AlgJ) in *P. aeruginosa* (Kristin E. Low et al., *JBC*, 2023).

3. Consider moving the working model from Figure S13 to the main text to improve clarity. Provide clearer axis labels, explicitly label SEC peaks (with theoretical molecular weights) in Figure 1, and add rotated 3D surface representations of the 12-mer and 24-mer cages to better illustrate pore architecture.

>>We thank the reviewer for kind suggestions.

We agree that including the working model in the main text would improve clarity. However, because this manuscript is being considered under the "Short Reports" format guidelines for *PLOS Biology*, we are limited to a maximum of four main figures. We have therefore retained the working model in the Supporting Information, where it has been updated and renumbered as Figure S14.

As requested by reviewer 3 as well, we have included the molecular weight standards in the analytical SEC profiles of Fig. 1 C and D and Fig. S1 A and B. The theoretical MW of different MucD oligomers is also mentioned in the related figure legends.

We have adjusted the surface representation of MucD and DegP cages in Fig. S6A to better illustrate the pore architectures. Given the high symmetry structures of MucD and DegP cages, a representative view along principal symmetry axes was presented in Fig. S6A.

4.Correct "EMBD" to "EMDB" and "18 A" to "18 Å".

>>We thank the reviewer for the careful inspection, and the mentioned typos have been corrected.


Reviewer #2: This study provides a comprehensive and significant advance in understanding the function of the HtrA-like serine protease MucD in Pseudomonas aeruginosa. The authors convincingly demonstrate that MucD directly degrades the alginate biosynthesis proteins AlgK and AlgX as physiological substrates, characterize its cleavage preferences via LC-MS/MS and bioinformatics, and determine high-resolution cryo-EM structures of substrate-bound MucD in 12-mer and 24-mer oligomeric states. These structures, combined with biochemical and mutational analyses, elegantly reveal that MucD exists in a dynamic equilibrium between a less-active 12-mer resting state and an active trimer, with substrate binding favoring the trimer and PDZ1 domain playing a key role in substrate engagement. Importantly, the work establishes a post-translational proteolytic mechanism for MucD-mediated negative regulation of alginate production, distinct from previously proposed transcriptional roles, offering valuable new insights into bacterial protein quality control and virulence regulation. The work is of high scientific quality and, after addressing the minor points below, is strongly recommended for publication.

>>Thank you for the positive comments.

Minor comments:
1. The Congo red assay (Lines 431-440) is unconventional for alginate quantification, as this dye primarily binds to cellulose, curli, or Psl/Pel polysaccharides. The authors should either provide validation that Congo red reliably correlates with alginate levels in their strain background, or supplement the data with a more established alginate-specific assay (e.g., uronic acid quantification).

>> We appreciate this valuable suggestion for the reviewer to improve the specificity of our phenotypic data. Please also see our response for reviewer 1, “Major Concerns” 4.

Following the reviewer's advice, we have tested the alginate production with the highly established carbazole assay for total uronic acid quantification. The quantitative results from the carbazole assay corroborate our Congo red assays, showing the same trends in alginate production among the strains.

We have updated Fig. 5 (now Fig. S12 due to number limits of main figures for a “Short Reports” type manuscript) and revised the corresponding “Results” section (Lines 425-427):

“…To determine whether this regulation depends on MucD protease activity, we measured alginate-producing levels in Δ*mucD* mutants in the PAO1 background using the carbazole assay, a quantitative method for detecting alginate in *P. aeruginosa* [19,32]…”

We also revised the related “Methods” section to describe the carbazole assay protocol accordingly.

2. Fig. 1 A-B: To further strengthen the characterization of MucD oligomeric states, we recommend performing Blue Native PAGE (BN-PAGE) or Native PAGE on the purified MucD protein.

>> We appreciate the reviewer's recommendation to validate the oligomeric states of MucD using a non-denaturing gel system. We performed native PAGE analysis of our purified MucD protein sample and two distinct bands were resolved, likely corresponding to the 12-mer (major population) and trimer (cannot determine molecular weight by native PAGE).

We have included this native PAGE result below for your review (Fig. R2) rather than adding it to the main text figures, given that the analytical SEC profiles (Fig. S1 A and B) have already provided high-resolution confirmation of these oligomeric states (clearly calibrated molecular weight) as well as we have limited space for quite a lot of figures.

**Figure R2. Native PAGE analysis of purified MucD protein.**

Purified wild-type MucD was resolved on a 3 to 12% NativePAGE™ Bis-Tris Gels (Invitrogen) and stained with Coomassie blue dye.

3. LC-MS controls: The degradation product mapping via UMSAP (Lines 145-156) does not mention negative controls (e.g., heat-inactivated MucD, S217A mutant, or substrate-only incubations) to rule out spontaneous hydrolysis or background peptide generation during the 8-hour incubation and concentration steps. Inclusion or discussion of these controls is recommended.

>> We appreciate the recommended control experiment from the reviewer.

To rule out these non-enzymatic factors, we performed the recommended control experiments by incubating the substrates (AlgK and AlgX) alone for the identical 8-hour duration and subjecting them to the exact same concentration and processing steps.

The LC-MS analysis of these substrate-only controls revealed minimal background degradation: only around 40 distinct peptides were detected, which accounted for ~15% of the total protein sequence coverage for each sample. In contrast, our experimental samples containing active MucD yielded extensive degradation profiles, covering nearly the entire protein sequence with hundreds of unique cleavage products detected (Fig. S3A). These results demonstrated that spontaneous hydrolysis or background processing during sample preparation was negligible and the vast majority of the mapped degradation products were MucD-dependent.

We have rephrased the “Results” section (Lines 153-156) and updated Fig. S3A accordingly:

“…The background peptides generation was negligible, as only 39 peptides matching AlgK (15.6% sequence coverage) and 38 peptides matching AlgX (14.3% sequence coverage) from substrate-only incubations were detected by LC-MS (Fig. S3A).”

4. Fig. S7: The sentence "The workflow for the 24-mer (A) and 12-mer (B) of MucD(S217A)-AlgK369-388 complex dataset processing and the local resolution estimation of the locally refined trimer electron density maps" is missing a period at the end.
>> We thank the reviewer for the careful inspection, and this typo has been corrected.


Reviewer #3: In the manuscript titled "Molecular basis of proteolytic regulation of alginate biosynthesis by HtrA-like protease MucD in Pseudomonas aeruginosa", Jiang et al. identify that MucD targets two novel substrates, AlgX and AlgK, which are involved in alginate biosynthesis to directly inhibit its production. The authors further characterize how the oligomerization state of MucD impacts substrate recognition and specificity through in vitro degradation assays and solving the first cryo-EM structure of AlgK bound to MucD. Overall, this work uses numerous biochemical and structure based mutational approaches to provide a substantial contribution to understanding of how MucD regulates alginate production. This study provides important insight into MucD mediate regulation of alginate biosynthesis. Below are listed major and minor concerns for this manuscript:
>> Thank you for the positive comments.

Major Concerns:
1. In Figures 1A, S1A, and S1B analytical size-exclusion chromatography profiles were used to analyze the molecular weight of the trimer and 12mer state of MucD, however there are no molecular weight standards on the graphs. Molecular weight standards should be included to better support the interpretation of the oligomeric state of MucD.

>>We thank the reviewer for pointing out this. To clarify our workflow, the SEC profile originally shown in Fig. 1A was generated using a preparative gel filtration column (HiLoad 16/600 Superdex 200 pg, Cytiva) for initial protein purification. The major two peaks from this purification step were then calibrated using a high-resolution analytical column (Superose 6 Increase 10/300 GL, Cytiva), respectively, as shown in Fig. S1 A and B.

Because the analytical Superose 6 column provides better separation resolution, we performed our column calibration and applied molecular weight standards for molecular weight determination specifically to this analytical setup. Accordingly, we have revised the analytical SEC profiles in Fig. 1 C and D, Fig. S1 A and B to incorporate the calibrated standard markers directly onto the profiles and updated the corresponding figure legends.

2. In Figure 1A, the authors claim that there are no proteins that co-elute with MucD based on SDS-PAGE and MALDI-TOF/MS suggesting that MucD lacks chaperone-like function. However, the gel shows a distinct band below the predominant MucD band suggesting that is could be either a MucD degradation product or a contaminating protein. In addition, there is no MALDI-TOF/MS data present in the manuscript to support that MucD is the sole protein present in the sample. Inclusion of MALDI-TOF/MS data in the manuscript should be included to conclude whether MucD has a chaperone-like function.

>>We appreciate the reviewer's careful inspection of the SDS-PAGE gel in Fig. 1A and the opportunity to clarify our mass spectrometry documentation.

The MALDI-TOF/MS identification results are now included in Table S1 of the supporting information. Given the low confidence and abundance of other background proteins apart from MucD based on the MS results, we believe the faint band noted by the reviewer should be a minor degradation fragment of MucD itself rather than an interacting partner. We have revised the text accordingly (Line 97) for this mass spectrometry verification.

Chaperone functions are highly condition-dependent (often triggered exclusively under specific environmental stresses, altered temperatures, or unique substrate states). In combination with the first reviewer's suggestion (reviewer 1, “Minor Concerns” 1), we have moderated our claims in the revised manuscript to avoid overstating that “MucD may lack chaperone-like function” (Lines 94-95):

“...Furthermore, no convincing additional protein co-eluting with MucD was detected based on SDS-PAGE (Fig. 1A) or MALDI-TOF/MS (Table S1) …”

3. Line 97 Can authors provide data to support dynamic equilibrium? Does isolation of the 12-mer and reapplication to gel filtration result in trimers and 12-mers?

>> We thank the reviewer for giving us a chance to clarify the workflow. When we isolated the fractions from the two main peaks after initial SEC purification (Fig. 1A) and re-applied them separately to the analytical gel filtration column, the protein re-equilibrated to yield distinct peaks for both the 12-mer and the trimer species (Fig. S1 A and B). We have rephrased this section (Lines 90–94) to clearly describe this isolation and re-injection workflow:

“…Isolation and re-application of two oligomeric populations to analytical SEC separately further identified them as a trimer and a 12-mer (main population), based on molecular weight (MW) calibration (Fig. S1 A and B). Analytical SEC also indicated that the MucD 12-mer and trimer exist in a dynamic equilibrium in solution.…”

4. Previous studies have shown that AlgX and AlgK form a complex with AlgJ and AlgF to produce the export complex required for biofilm formation (Low et al, JBC 2023). The authors provide substantial evidence that MucD targets AlgX and AlgK for degradation, but it is unclear whether MucD could also target other proteins in this complex or other proteins involved in alginate biosynthesis. Inclusion of a degradation assay with MucD and AlgJ or AlgF would be informative to understand substrate specificity of MucD.
>> Thanks for the reviewer’s constructive suggestion and we totally agree that exploring whether MucD targets the remaining members of the AlgF-AlgJ-AlgK-AlgX export complex (such as AlgJ or AlgF) is highly informative for understanding its overall substrate specificity.

To address this, we conducted *in vitro* degradation assays by incubating MucD with purified soluble constructs of AlgF_30-216_ or AlgJ_79-379_. We have added the new data to the supporting information (Fig. S13) and incorporated a corresponding description in “Results” section of the revised manuscript (Lines 457–463):

“…Previous studies demonstrated that alginate export and modification require a large complex composed of AlgK, AlgX, AlgJ, and AlgF in *P. aeruginosa* [37]. To test whether MucD also targets other alginate synthesis proteins in this complex, we purified soluble constructs of AlgJ_79-379_ and AlgF_30-216_ [37,38] and examined their susceptibility to MucD-mediated degradation. Interestingly, AlgF was degraded by MucD, whereas AlgJ was resistant (Fig. S13 A and B), suggesting that post-translational regulation by MucD is selective and restricted to particular components of the alginate biosynthetic machinery…”

5. Can authors please provide a non-specific substrate control?

>> As described in the previous response, our results revealed that AlgJ was resistant to MucD-mediated degradation (Fig. S13A), which can act as a non-specific substrate control.

6. What provides specificity for the protease? Authors mention "F236 in MucD forms a hydrophobic interaction with L372 of the bound AlgK369-388, and it could enable a potential π-π interaction with aromatic amino acids in some substrates to enhance binding. Furthermore, R238 in MucD forms a salt bridge with D370 of AlgK369-388, which significantly increases the capacity of the L2 to contact the negatively charged residues of the potential substrates" can they please test these interfaces for importance? For potential π-π interaction and salt bridge, what are distances between residues. Please support this hypothesis by mutating MucD F236 or R238 and test for activity with time of digestion and concentration of substrate?

>>We thank the reviewer for this constructive suggestion to experimentally validate the structural interfaces driving MucD substrate specificity.

We measured the specific atomic distances within our cryo-EM structural model. The distance between the sidechain guanidinium group of MucD-R238 and the carboxylate group of AlgK-D370 is ~3.4 Å, which is well within the classic range for a salt bridge. Additionally, the distance between the aromatic ring of MucD-F236 and the side chain of AlgK_369-388_-L372 is ~3.9 Å, supporting the formation of localized hydrophobic interactions.

To test the functional importance of these interfaces, we successfully generated a MucD(F236A) mutant and tested its proteolytical activity. We have included the new functional data in the Supporting Information (Fig. S9 E and F) and updated “Results” section of the revised manuscript accordingly (Lines 296–300):

“…Consistent with this role, substitution of F236 with alanine markedly reduced MucD proteolytical activity without disrupting oligomeric equilibrium: only limited AlgK degradation by MucD(F236A) was observed after 4 hours of incubation (Fig. S9 E and F, Fig. 1B, Fig. S1C). These results indicate that F236-mediated hydrophobic interaction is important for substrate recognition.”

7. Figures 4D, 4E, S5B and S11E wild-type MucD - AlgK degradation control missing.

>> We thank the reviewer for pointing out the importance of degradation controls. The degradation controls for wild-type MucD against AlgK have been characterized and presented in Fig. 1B and Fig. S1C. Due to space limitations in the multi-panel layouts of Figures 4D, 4E, S5B, and S11E, we did not repeat the identical control and instead focus on comparing between MucD variants.

We have updated citations of Fig. 4D, 4E, S5B and S11E in the Main Text to cross-reference the wild-type data in Fig. 1B and Fig. S1C for the reader's convenience (Lines 368-369, 386-388, 231-233 and 325-327).

8. Figure 4D and E, authors claim that partially degraded AlgK was identified by MALDI-MS but no data is present except for SDS-PAGE gel and SEC chromatograph.

>> The MALDI-TOF/MS data identifying the partially degraded AlgK fragments is now provided in Table S3 of the Supporting Information and referenced in the main text (Line 368).

9. Figure S5E, in the SDS-PAGE gel data mention that MucD is S217A mutant.
>> The labels of the mentioned SDS-PAGE gel in Fig. S5E have been corrected.

10. Figure 5A provides convincing data regarding alginate production in the ΔmucD and complemented strains. However, the authors claim that "the reduction in alginate production in not due to differences in cell fitness" and use an endpoint OD600 reading to support this. A single time-point at the end of the bacterial growth cycle does not provide sufficient information to claim that alginate production does not impact cell fitness. A 24-hour growth curve should be provided for each strain to test whether cell fitness is impacted.
>>We thank the reviewer for this constructive recommendation to more rigorously assess cell fitness. Following the suggestion, we determined the 24-hour growth curve analysis for all strains tested for alginate production under the same condition. The data revealed no notable differences in cell fitness of the PAO1, Δ*mucD* and complemented strains.

We have included these growth curves in the Supporting Information (Fig. S12B) and updated the “Results” section (Lines 432-433) accordingly:

“…Growth monitoring over 24 hours confirmed that these differences were not caused by changes in cell fitness (Fig. S12B)…”

11. In Figure 5B the authors claim there is no significant changes in the algK and algX mRNA levels between the wild-type and ΔmucD strains, however previous studies have reported a significant increase in promoter activity for other alg genes in the ΔmucD strain. This qPCR data could be strengthened by including a positive control such as algD and including a complementation strain. Additionally, a complementation strain should be used in Figure 5C as a control.

>> We thank the reviewer for this insightful comment, please also see our response for reviewer 1, “Major Concerns” 5.

To address this, we examined the mRNA levels of *algK*, *algX* and *algD* by quantitative reverse transcription PCR (RT-qPCR) in *mucD* related variants. While *algK* and *algX* mRNA levels remained unchanged, the transcription of *algD*—the first gene encoding the rate-limiting enzyme in the operon—was significantly increased in the Δ*mucD* strain (Fig. S12C). We have revised the “Result” section accordingly to address these findings and provide possible explanations (Lines 438-448):

“A previous study reported that deletion of *mucD* in PAO1 increases promoter activity of the *alg* operon [14]. To assess whether the elevated alginate production observed here was accompanied by transcriptional changes in *algK and algX*, quantitative reverse transcription PCR (RT-qPCR) for the wild-type, Δ*mucD,* and Δ*mucD*::*mucD* strains was carried out. The results showed that the mRNA levels of *algK* and *algX* did not differ significantly among the strains. By contrast, *algD*, the proximal gene in the operon encoding the rate-limiting enzyme of the pathway, increased ~2.8-fold in the Δ*mucD* strain and restored to wild-type levels upon *mucD* complementation (Fig. S12C). The different behavior of the proximal *algD* transcript and the distal *algK* and *algX* transcripts likely reflects natural transcriptional attenuation or differential mRNA stability across this large 12-gene biosynthetic cluster, consistent with previous evidence for increased promoter activity in Δ*mucD* cells [14,35,36]…”

We have also revised the “Discussion” section (Lines 551-556) to highlight this dual-layer regulation:

“…In large bacterial operons such as the *alg* operon, polycistronic transcripts can undergo progressive downstream attenuation, internal termination, or segmented mRNA decay, uncoupling steady-state transcript levels of distal structural genes (*algK* and *algX*) from primary promoter activity [34]. Collectively, these findings indicate that MucD regulates alginate biosynthesis through both negative transcriptional control of the *alg* operon and post-translational quality control of key assembly components, including AlgK and AlgX…”

Following the reviewer’s suggestion, we have also included the complementation strain Δ*mucD*::*mucD* for detection of AlgK protein level as a control (Fig. S12D) and updated the “Results” section accordingly (Lines 448-451):

“…Given that the AlgK is a MucD substrate *in vitro*, we next examined AlgK protein level *in vivo* by Western blot in PAO1, Δ*mucD*, and Δ*mucD*::*mucD* strains. Our results revealed that AlgK protein level was markedly increased in the Δ*mucD* mutant and restored by *mucD* complementation (Fig. S12D)…”

The results support our main conclusion of the post-translational regulation role of MucD in alginate biosynthesis.

Minor Concerns:
1. In lines 50-52 for clarity spell out what PDZ stands for then use the abbreviation and remove the text "an initialism combining the first letters of the first three proteins discovered to share the domain".

>> Following the suggestion, the mentioned text (Lines 46-48) have been revised to directly define the domain acronym as follows:

“…one or two PDZ (**p**ost synaptic density protein 95 (PSD95), *Drosophila* **d**isc large tumor suppressor (Dlg1), and **z**onula occludens-1 protein (zo-1)) domains [7]..."

2. Line 87 "defences" should be revised to say "defenses".

>>We thank the reviewer for the careful inspection, and the typo has been corrected.

3. Lines 495 and 528 "chaperon" should be revised to say "chaperone"

>> We thank the reviewer for the careful inspection, and the typos have been corrected.
