# Supplementary material for: MucD regulates alginate biosynthesis through the proteolytic control of AlgX and AlgK in Pseudomonas aeruginosa
Source: PLoS Biol. 2026 Sep 21;24(9):e3003985. doi: 10.1371/journal.pbio.3003985 (PMC13608001; doi:10.1371/journal.pbio.3003985)
Supplement: Peer review history

# Editor report: Decision Letter 0

**DOI:** 10.1371/journal.pbio.3003985.r001

**Authors:** Richard Hodge

5 May 2026

Dear Dr Gao,

Thank you for submitting your manuscript entitled "Molecular basis of proteolytic regulation of alginate biosynthesis by HtrA-like protease MucD in Pseudomonas aeruginosa" for consideration as a Research Article by PLOS Biology. Please accept my sincere apologies for the delay in getting back to you as we consulted with an academic editor about your submission.

Your manuscript has now been evaluated by the PLOS Biology editorial staff, as well as by an academic editor with relevant expertise, and I am writing to let you know that we would like to send your submission out for external peer review.

IMPORTANT - After discussions within the editorial team, we would like to consider your manuscript as a Short Report (https://journals.plos.org/plosbiology/s/what-we-publish#loc-short-reports). Upon resubmission (see guidelines below), I would be grateful if you could please tick 'Short Report' as the article type in the dropdown menu in the online submission form.

Before we can send your manuscript to reviewers, we need you to complete your submission by providing the metadata that is required for full assessment. To this end, please login to Editorial Manager where you will find the paper in the 'Submissions Needing Revisions' folder on your homepage. Please click 'Revise Submission' from the Action Links and complete all additional questions in the submission questionnaire.

Once your full submission is complete, your paper will undergo a series of checks in preparation for peer review. After your manuscript has passed the checks it will be sent out for review. To provide the metadata for your submission, please Login to Editorial Manager (https://www.editorialmanager.com/pbiology) within two working days, i.e. by May 07 2026 11:59PM.

If your manuscript has been previously peer-reviewed at another journal, PLOS Biology is willing to work with those reviews in order to avoid re-starting the process. Submission of the previous reviews is entirely optional and our ability to use them effectively will depend on the willingness of the previous journal to confirm the content of the reports and share the reviewer identities. Please note that we reserve the right to invite additional reviewers if we consider that additional/independent reviewers are needed, although we aim to avoid this as far as possible. In our experience, working with previous reviews does save time.

If you would like us to consider previous reviewer reports, please edit your cover letter to let us know and include the name of the journal where the work was previously considered and the manuscript ID it was given. In addition, please upload a response to the reviews as a 'Prior Peer Review' file type, which should include the reports in full and a point-by-point reply detailing how you have or plan to address the reviewers' concerns.

During the process of completing your manuscript submission, you will be invited to opt-in to posting your pre-review manuscript as a bioRxiv preprint. Visit http://journals.plos.org/plosbiology/s/preprints for full details. If you consent to posting your current manuscript as a preprint, please upload a single Preprint PDF.

Feel free to email us at plosbiology@plos.org if you have any queries relating to your submission.

Kind regards,

Richard

Richard Hodge, PhD

Senior Editor, PLOS Biology

rhodge@plos.org

PLOS

Empowering researchers to transform science

Carlyle House, Carlyle Road, Cambridge, CB4 3DN, United Kingdom

California (U.S.) corporation #C2354500, based in San Francisco

# Peer review: Decision Letter 1

**DOI:** 10.1371/journal.pbio.3003985.r002

**Authors:** Richard Hodge

3 Jun 2026

Dear Dr Gao,

Thank you for your patience while your manuscript "Molecular basis of proteolytic regulation of alginate biosynthesis by HtrA-like protease MucD in Pseudomonas aeruginosa" was peer-reviewed at PLOS Biology. It has been evaluated by the PLOS Biology editors, an Academic Editor with relevant expertise, and by three independent reviewers.

As you will see in the reviewer reports, which can be found at the end of this email, although the reviewers find the work potentially interesting, they have also raised a substantial number of important concerns. Based on their specific comments and following discussion with the Academic Editor, it is clear that a substantial amount of work would be required to meet the criteria for publication in PLOS Biology. However, given our and the reviewer interest in your study, we would be open to inviting a comprehensive revision of the study that thoroughly addresses all the reviewers' comments. Given the extent of revision that would be needed, we cannot make a decision about publication until we have seen the revised manuscript and your response to the reviewers' comments. Your revised manuscript would need to be seen by the reviewers again, but please note that we would not engage them unless their main concerns have been addressed.

The reviewers think that the manuscript is interesting and that the structural aspects are well done, but they raise overlapping concerns about the overall strength of the physiological validation of the structural models. Specifically, Reviewer #1 notes that additional in vivo evidence is required to demonstrated that AlgK and AlgX are physiological substrates and that the MucD 12-mer mutant is tested in alginate complementation assays. Reviewer #2 raises concerns with the Congo red assay used to quantify alginate and request that orthogonal validation is provided. Reviewer #3 notes that additional experiments are required to understand the substrate specificity (using MucD interface mutants and degradation assays with AlgJ/AlgF), to test how cell fitness is impacted in the mucD strains and notes that important controls are missing in several experiments.

In addition to these revisions, you will need to complete some formatting changes, which you will receive in a follow up email. A member of our team will be in touch with a set of requests shortly.

We appreciate that these requests represent a great deal of extra work, and we are willing to relax our standard revision time to allow you 6 months to revise your study. Please email us (plosbiology@plos.org) if you have any questions or concerns, or envision needing a (short) extension.

At this stage, your manuscript remains formally under active consideration at our journal; please notify us by email if you do not intend to submit a revision so that we may withdraw it.

**IMPORTANT - SUBMITTING YOUR REVISION**

Your revisions should address the specific points made by each reviewer. Please submit the following files along with your revised manuscript:

1. A 'Response to Reviewers' file - this should detail your responses to the editorial requests, present a point-by-point response to all of the reviewers' comments, and indicate the changes made to the manuscript.

*NOTE: In your point by point response to the reviewers, please provide the full context of each review. Do not selectively quote paragraphs or sentences to reply to. The entire set of reviewer comments should be present in full and each specific point should be responded to individually, point by point.

You should also cite any additional relevant literature that has been published since the original submission and mention any additional citations in your response.

2. In addition to a clean copy of the manuscript, please also upload a 'track-changes' version of your manuscript that specifies the edits made. This should be uploaded as a "Revised Article with Changes Highlighted " file type.

*Resubmission Checklist*

When you are ready to resubmit your revised manuscript, please refer to this resubmission checklist: https://plos.io/Biology_Checklist

To submit a revised version of your manuscript, please go to https://www.editorialmanager.com/pbiology/ and log in as an Author. Click the link labelled 'Submissions Needing Revision' where you will find your submission record.

Please make sure to read the following important policies and guidelines while preparing your revision:

*Published Peer Review*

Please note while forming your response, if your article is accepted, you may have the opportunity to make the peer review history publicly available. The record will include editor decision letters (with reviews) and your responses to reviewer comments. If eligible, we will contact you to opt in or out. Please see here for more details:

https://blogs.plos.org/plos/2019/05/plos-journals-now-open-for-published-peer-review/

*PLOS Data Policy*

Please note that as a condition of publication PLOS' data policy (http://journals.plos.org/plosbiology/s/data-availability) requires that you make available all data used to draw the conclusions arrived at in your manuscript. If you have not already done so, you must include any data used in your manuscript either in appropriate repositories, within the body of the manuscript, or as supporting information (N.B. this includes any numerical values that were used to generate graphs, histograms etc.). For an example see here: http://www.plosbiology.org/article/info%3Adoi%2F10.1371%2Fjournal.pbio.1001908#s5

*Blot and Gel Data Policy*

We require the original, uncropped and minimally adjusted images supporting all blot and gel results reported in an article's figures or Supporting Information files. We will require these files before a manuscript can be accepted so please prepare them now, if you have not already uploaded them. Please carefully read our guidelines for how to prepare and upload this data: https://journals.plos.org/plosbiology/s/figures#loc-blot-and-gel-reporting-requirements

*Protocols deposition*

To enhance the reproducibility of your results, we recommend that if applicable you deposit your laboratory protocols in protocols.io, where a protocol can be assigned its own identifier (DOI) such that it can be cited independently in the future. Additionally, PLOS ONE offers an option for publishing peer-reviewed Lab Protocol articles, which describe protocols hosted on protocols.io. Read more information on sharing protocols at https://plos.org/protocols?utm_medium=editorial-email&utm_source=authorletters&utm_campaign=protocols

Thank you again for your submission to our journal. We hope that our editorial process has been constructive thus far, and we welcome your feedback at any time. Please don't hesitate to contact us if you have any questions or comments.

Best regards,

Richard

Richard Hodge, PhD

Senior Editor, PLOS Biology

rhodge@plos.org

-----------------------------------------

REVIEWS:

Reviewer #1: This manuscript presents a structural and biochemical investigation of the HtrA-like protease MucD in Pseudomonas aeruginosa. The authors identified AlgK and AlgX as direct substrates, determined cryo-EM structures of MucD assemblies, and proposed a novel post-translational regulatory mechanism for alginate biosynthesis governed by a trimer-to-12-mer dynamic equilibrium. While the structural biology work is substantial and uncovers an intriguing mechanism distinct from its homolog DegP , the manuscript currently lacks the robust in vivo physiological validation. Specifically, the physiological relevance of the proposed structural models and the in vivo substrate verification require further experimental support. I recommend a Major Revision pending the inclusion of critical supplemental experiments.

Major Concerns

1. The claim that AlgK and AlgX are physiological substrates relies heavily on in vitro assays. In vivo evidence is currently limited to an increase in AlgK abundance in the ΔmucD mutant. The authors must provide direct in vivo interaction evidence (e.g., co-IP, in-cell crosslinking, or substrate trapping with the S217A mutant) and examine AlgX protein levels in vivo.

2. The authors engineered a disulfide-locked 12-mer mutant, MucD(D282C-T469C), demonstrating its reduced in vitro activity. To form a complete biological loop, this specific mutant must be tested in the in vivo alginate complementation assay. If the structural model holds true, this locked resting-state mutant should fail to suppress alginate overproduction.

3. A significant discrepancy exists regarding oligomerization: full-length substrates induce the active trimer , whereas the synthetic short peptide (AlgK369-388) induces a 24-mer, which was subsequently used for cryo-EM. The authors must clarify and discuss whether this peptide-bound 24-mer represents an authentic physiological recognition event or a post-cleavage intermediate/artifact.

4. The Congo red assay used to measure EPS is non-specific and can bind to other extracellular polysaccharides (Pel, Psl) and matrix components. To robustly support the central physiological conclusion, the authors must utilize an orthogonal, alginate-specific assay.

5. The conclusion that MucD represses alginate production primarily via post-translational degradation, independent of transcription, is overstated. Unchanged algK/algX mRNA levels do not preclude transcriptional regulation of other operon genes (such as the rate-limiting enzyme algD) or broader AlgU-dependent effects. The authors should moderate these claims and comprehensively discuss potential crosstalk between these pathways.

6. The proposed substrate-induced 12-mer-to-trimer conversion relies primarily on SEC analysis, which reflects equilibrium states rather than dynamic transitions. The authors are encouraged to provide quantitative enzymatic analyses to strengthen the rigor of this conclusion.

Minor Concerns

1. The assertion that MucD lacks chaperone activity based solely on the absence of co-purified proteins is unconvincing, as such activity is highly condition-dependent. This statement should be softened or revised.

2. Figure 5C lacks densitometric quantification. Furthermore, utilizing a cytoplasmic protein as a loading control for periplasmic proteins may not account for extraction efficiency differences or periplasmic-specific leakage. Please justify this choice or use a periplasmic control.

3. Consider moving the working model from Figure S13 to the main text to improve clarity. Provide clearer axis labels, explicitly label SEC peaks (with theoretical molecular weights) in Figure 1, and add rotated 3D surface representations of the 12-mer and 24-mer cages to better illustrate pore architecture.

4.Correct "EMBD" to "EMDB" and "18 A" to "18 Å".

Reviewer #2: This study provides a comprehensive and significant advance in understanding the function of the HtrA-like serine protease MucD in Pseudomonas aeruginosa. The authors convincingly demonstrate that MucD directly degrades the alginate biosynthesis proteins AlgK and AlgX as physiological substrates, characterize its cleavage preferences via LC-MS/MS and bioinformatics, and determine high-resolution cryo-EM structures of substrate-bound MucD in 12-mer and 24-mer oligomeric states. These structures, combined with biochemical and mutational analyses, elegantly reveal that MucD exists in a dynamic equilibrium between a less-active 12-mer resting state and an active trimer, with substrate binding favoring the trimer and PDZ1 domain playing a key role in substrate engagement. Importantly, the work establishes a post-translational proteolytic mechanism for MucD-mediated negative regulation of alginate production, distinct from previously proposed transcriptional roles, offering valuable new insights into bacterial protein quality control and virulence regulation. The work is of high scientific quality and, after addressing the minor points below, is strongly recommended for publication.

Minor comments:

1.The Congo red assay (Lines 431-440) is unconventional for alginate quantification, as this dye primarily binds to cellulose, curli, or Psl/Pel polysaccharides. The authors should either provide validation that Congo red reliably correlates with alginate levels in their strain background, or supplement the data with a more established alginate-specific assay (e.g., uronic acid quantification).

2.Fig. 1 A-B: To further strengthen the characterization of MucD oligomeric states, we recommend performing Blue Native PAGE (BN-PAGE) or Native PAGE on the purified MucD protein.

3.LC-MS controls: The degradation product mapping via UMSAP (Lines 145-156) does not mention negative controls (e.g., heat-inactivated MucD, S217A mutant, or substrate-only incubations) to rule out spontaneous hydrolysis or background peptide generation during the 8-hour incubation and concentration steps. Inclusion or discussion of these controls is recommended.

4.Fig. S7: The sentence "The workflow for the 24-mer (A) and 12-mer (B) of MucD(S217A)-AlgK369-388 complex dataset processing and the local resolution estimation of the locally refined trimer electron density maps" is missing a period at the end.

Reviewer #3: In the manuscript titled "Molecular basis of proteolytic regulation of alginate biosynthesis by HtrA-like protease MucD in Pseudomonas aeruginosa", Jiang et al. identify that MucD targets two novel substrates, AlgX and AlgK, which are involved in alginate biosynthesis to directly inhibit its production. The authors further characterize how the oligomerization state of MucD impacts substrate recognition and specificity through in vitro degradation assays and solving the first cryo-EM structure of AlgK bound to MucD. Overall, this work uses numerous biochemical and structure based mutational approaches to provide a substantial contribution to understanding of how MucD regulates alginate production. This study provides important insight into MucD mediate regulation of alginate biosynthesis. Below are listed major and minor concerns for this manuscript:

Major Concerns:

1.In Figures 1A, S1A, and S1B analytical size-exclusion chromatography profiles were used to analyze the molecular weight of the trimer and 12mer state of MucD, however there are no molecular weight standards on the graphs. Molecular weight standards should be included to better support the interpretation of the oligomeric state of MucD.

2.In Figure 1A, the authors claim that there are no proteins that co-elute with MucD based on SDS-PAGE and MALDI-TOF/MS suggesting that MucD lacks chaperone-like function. However, the gel shows a distinct band below the predominant MucD band suggesting that is could be either a MucD degradation product or a contaminating protein. In addition, there is no MALDI-TOF/MS data present in the manuscript to support that MucD is the sole protein present in the sample. Inclusion of MALDI-TOF/MS data in the manuscript should be included to conclude whether MucD has a chaperone-like function.

3.Line 97 Can authors provide data to support dynamic equilibrium? Does isolation of the 12-mer and reapplication to gel filtration result in trimers and 12-mers?

4.Previous studies have shown that AlgX and AlgK form a complex with AlgJ and AlgF to produce the export complex required for biofilm formation (Low et al, JBC 2023). The authors provide substantial evidence that MucD targets AlgX and AlgK for degradation, but it is unclear whether MucD could also target other proteins in this complex or other proteins involved in alginate biosynthesis. Inclusion of a degradation assay with MucD and AlgJ or AlgF would be informative to understand substrate specificity of MucD.

5.Can authors please provide a non-specific substrate control?

6.What provides specificity for the protease? Authors mention "F236 in MucD forms a hydrophobic interaction with L372 of the bound AlgK369-388, and it could enable a potential π-π interaction with aromatic amino acids in some substrates to enhance binding. Furthermore, R238 in MucD forms a salt bridge with D370 of AlgK369-388, which significantly increases the capacity of the L2 to contact the negatively charged residues of the potential substrates" can they please test these interfaces for importance? For potential π-π interaction and salt bridge, what are distances between residues. Please support this hypothesis by mutating MucD F236 or R238 and test for activity with time of digestion and concentration of substrate?

7.Figures 4D, 4E, S5B and S11E wild-type MucD - AlgK degradation control missing.

8.Figure 4D and E, authors claim that partially degraded AlgK was identified by MALDI-MS but no data is present except for SDS-PAGE gel and SEC chromatograph.

9.Figure S5E, in the SDS-PAGE gel data mention that MucD is S217A mutant.

10.Figure 5A provides convincing data regarding alginate production in the ΔmucD and complemented strains. However, the authors claim that "the reduction in alginate production in not due to differences in cell fitness" and use an endpoint OD600 reading to support this. A single time-point at the end of the bacterial growth cycle does not provide sufficient information to claim that alginate production does not impact cell fitness. A 24-hour growth curve should be provided for each strain to test whether cell fitness is impacted.

11.In Figure 5B the authors claim there is no significant changes in the algK and algX mRNA levels between the wild-type and ΔmucD strains, however previous studies have reported a significant increase in promoter activity for other alg genes in the ΔmucD strain. This qPCR data could be strengthened by including a positive control such as algD and including a complementation strain. Additionally, a complementation strain should be used in Figure 5C as a control.

Minor Concerns:

1.In lines 50-52 for clarity spell out what PDZ stands for then use the abbreviation and remove the text "an initialism combining the first letters of the first three proteins discovered to share the domain".

2.Line 87 "defences" should be revised to say "defenses".

3.Lines 495 and 528 "chaperon" should be revised to say "chaperone".

# Author comment: Author response to Decision Letter 2

**DOI:** 10.1371/journal.pbio.3003985.r003

15 Jul 2026

Attachment Submitted filename: MucD_Point_To_Point_Response_YGAO.docx

# Peer review: Decision Letter 2

**DOI:** 10.1371/journal.pbio.3003985.r004

**Authors:** Richard Hodge

14 Aug 2026

Dear Dr Gao,

Thank you for your patience while we considered your revised manuscript "Molecular basis of proteolytic regulation of alginate biosynthesis by HtrA-like protease MucD in Pseudomonas aeruginosa" for publication as a Short Report at PLOS Biology. This revised version of your manuscript has been evaluated by the PLOS Biology editors, the Academic Editor and the original reviewers.

Based on the reviews, I am pleased to say that we are likely to accept this manuscript for publication, provided you satisfactorily address the following data and other policy-related requests that I have provided below (A-H). We also encourage you to provide a schematic model as requested by Reviewer #2:

(A) We routinely suggest changes to titles to ensure maximum accessibility for a broad, non-specialist readership. In this case, we would suggest a couple of minor edits to the title for flow and additional information, as follows. Please ensure you change both the manuscript file and the online submission system, as they need to match for final acceptance:

“MucD regulates alginate biosynthesis through the proteolytic control of AlgX and AlgK in Pseudomonas aeruginosa”

(B) When providing a schematic model in response to Reviewer #2, please do not generate a new Figure 5, since your manuscript is being considered as a Short Report which have a maximum of 4 main figures. Please incorporate the model into the existing Figure 4 or include in the supplementary figures.

(C) You may be aware of the PLOS Data Policy, which requires that all data be made available without restriction: http://journals.plos.org/plosbiology/s/data-availability. For more information, please also see this editorial: http://dx.doi.org/10.1371/journal.pbio.1001797

Note that we do not require all raw data. Rather, we ask that all individual quantitative observations that underlie the data summarized in the figures and results of your paper be made available in one of the following forms:

-Supplementary files (e.g., excel). Please ensure that all data files are uploaded as 'Supporting Information' and are invariably referred to (in the manuscript, figure legends, and the Description field when uploading your files) using the following format verbatim: S1 Data, S2 Data, etc. Multiple panels of a single or even several figures can be included as multiple sheets in one excel file that is saved using exactly the following convention: S1_Data.xlsx (using an underscore).

-Deposition in a publicly available repository. Please also provide the accession code or a reviewer link so that we may view your data before publication.

Regardless of the method selected, please ensure that you provide the individual numerical values that underlie the summary data displayed in the following figure panels as they are essential for readers to assess your analysis and to reproduce it:

Figure 1A, 1C-D, 4B-C, S1A-B, S2A, S5A, S5C-F, S9E, S11D, S12A-D

NOTE: the numerical data provided should include all replicates AND the way in which the plotted mean and errors were derived (it should not present only the mean/average values).

(D) Thank you for providing the structural data in the PDB database (24XX, 22KG, 24YE, 22SK). However, we note that the data is currently on hold for release. We ask that you please make the structures publicly available at this stage before publication.

(E) In addition, please ensure that your Data Statement in the submission system accurately describes where the structures can be found and is in final format, as it will be published as written there.

(F) Please also ensure that each of the relevant figure legends in your manuscript include information on *WHERE THE UNDERLYING DATA CAN BE FOUND*, and ensure your supplemental data file/s has a legend.

(G) We require the original, uncropped and minimally adjusted images supporting all blot and gel results reported in the following Figures:

Figure 1A-B, 4D-E, S1C-E, S2A-B, S5A-B, S5E, S9F, S11E, S12D, S13A-B

We will require these files before a manuscript can be accepted so please prepare and upload them now. Please carefully read our guidelines for how to prepare and upload this data: https://journals.plos.org/plosbiology/s/figures#loc-blot-and-gel-reporting-requirements.

(H) Per journal policy, if you have generated any custom code during the course of this investigation, please make it available without restrictions. Please ensure that the code is sufficiently well documented and reusable, and that your Data Statement in the Editorial Manager submission system accurately describes where your code can be found. More information on our Code Policy, what and how to share can be found here: https://journals.plos.org/plosbiology/s/code-availability

Please note that we cannot accept sole deposition of code in GitHub, as this could be changed after publication. However, you can archive this version of your publicly available GitHub code to Zenodo. Once you do this, it will generate a DOI number, which you will need to provide in the Data Accessibility Statement (you are welcome to also provide the GitHub access information). See the process for doing this here: https://docs.github.com/en/repositories/archiving-a-github-repository/referencing-and-citing-content

------------------------------------------------------------------------

As you address these items, please take this last chance to review your reference list to ensure that it is complete and correct. If you have cited papers that have been retracted, please include the rationale for doing so in the manuscript text, or remove these references and replace them with relevant current references. Any changes to the reference list should be mentioned in the cover letter that accompanies your revised manuscript.

In addition to these revisions, you may need to complete some formatting changes, which you will receive in a follow up email. A member of our team will be in touch with a set of requests shortly. If you do not receive a separate email within a few days, please assume that checks have been completed, and no additional changes are required.

We expect to receive your revised manuscript within two weeks.

To submit your revision, please go to https://www.editorialmanager.com/pbiology/ and log in as an Author. Click the link labelled 'Submissions Needing Revision' to find your submission record. Your revised submission must include the following:

- a cover letter that should detail your responses to any editorial requests, if applicable, and whether changes have been made to the reference list

- a Response to Reviewers file that provides a detailed response to the reviewers' comments (if applicable, if not applicable please do not delete your existing 'Response to Reviewers' file.)

- a track-changes file indicating any changes that you have made to the manuscript.

NOTE: If Supporting Information files are included with your article, note that these are not copyedited and will be published as they are submitted. Please ensure that these files are legible and of high quality (at least 300 dpi) in an easily accessible file format. For this reason, please be aware that any references listed in an SI file will not be indexed. For more information, see our Supporting Information guidelines:

https://journals.plos.org/plosbiology/s/supporting-information

*Published Peer Review History*

Please note that you may have the opportunity to make the peer review history publicly available. The record will include editor decision letters (with reviews) and your responses to reviewer comments. If eligible, we will contact you to opt in or out. Please see here for more details:

https://plos.org/published-peer-review-history/

*Press*

Should you, your institution's press office or the journal office choose to press release your paper, please ensure you have opted out of Early Article Posting on the submission form. We ask that you notify us as soon as possible if you or your institution is planning to press release the article.

*Protocols deposition*

To enhance the reproducibility of your results, we recommend that if applicable you deposit your laboratory protocols in protocols.io, where a protocol can be assigned its own identifier (DOI) such that it can be cited independently in the future. Additionally, PLOS ONE offers an option for publishing peer-reviewed Lab Protocol articles, which describe protocols hosted on protocols.io. Read more information on sharing protocols at https://plos.org/protocols?utm_medium=editorial-email&utm_source=authorletters&utm_campaign=protocols

Please do not hesitate to contact me should you have any questions.

Best regards,

Richard

Richard Hodge, PhD

Senior Editor, PLOS Biology

rhodge@plos.org

-----------------------------------------------------------------------

Reviewer remarks:

Reviewer #1 (Rui Bao, identifies himself): In my view, the authors have addressed the major concerns satisfactorily. The revised manuscript provides a substantially clearer and more complete account of the biochemical, structural, and physiological evidence supporting the role of MucD in post-translational regulation of alginate biosynthesis. I do not have further substantive criticisms. I consider the manuscript suitable for publication in its present form.

Reviewer #2 (Yihua Huang, identifies himself): The authors fully addressed my concerns. For clarification, I suggest that the authors added the proteins AlgK, AlgX and MucD into a model of the proposed multiprotein complex in alginate biosynthesis in a supplementary figure.

Reviewer #3: The manuscript is now acceptable.

# Author comment: Author response to Decision Letter 3

**DOI:** 10.1371/journal.pbio.3003985.r005

18 Aug 2026

Attachment Submitted filename: MucD_Point_To_Point_Response_YGAO_auresp_3.docx

# Editor report: Decision Letter 3

**DOI:** 10.1371/journal.pbio.3003985.r006

**Authors:** Richard Hodge

19 Aug 2026

Dear Yong-Gui,

On behalf of my colleagues and the Academic Editor, Ann Stock, I am pleased to say that we can accept your manuscript for publication, provided you address any remaining formatting and reporting issues. These will be detailed in an email you should receive within 2-3 business days from our colleagues in the journal operations team; no action is required from you until then. Please note that we will not be able to formally accept your manuscript and schedule it for publication until you have completed any requested changes.

Please take a minute to log into Editorial Manager at http://www.editorialmanager.com/pbiology/, click the "Update My Information" link at the top of the page, and update your user information to ensure an efficient production process.

PRESS

We frequently collaborate with press offices. If your institution or institutions have a press office, please notify them about your upcoming paper at this point, to enable them to help maximise its impact. If the press office is planning to promote your findings, we would be grateful if they could coordinate with biologypress@plos.org. If you have previously opted in to the early version process, we ask that you notify us immediately of any press plans so that we may opt out on your behalf.

We also ask that you take this opportunity to read our Embargo Policy regarding the discussion, promotion and media coverage of work that is yet to be published by PLOS. As your manuscript is not yet published, it is bound by the conditions of our Embargo Policy. Please be aware that this policy is in place both to ensure that any press coverage of your article is fully substantiated and to provide a direct link between such coverage and the published work. For full details of our Embargo Policy, please visit http://www.plos.org/about/media-inquiries/embargo-policy/.

Thank you again for choosing PLOS Biology for publication and supporting Open Access publishing. We look forward to publishing your study.

Best wishes,

Richard

Richard Hodge, PhD

Senior Editor, PLOS Biology

rhodge@plos.org

PLOS

Empowering researchers to transform science

Carlyle House, Carlyle Road, Cambridge, CB4 3DN, United Kingdom

California (U.S.) corporation #C2354500, based in San Francisco
